# Cost–benefit analysis of the mechanisms that enable migrating cells to sustain motility upon changes in matrix environments

**DOI:** 10.1098/rsif.2014.1355

**Published:** 2015-05-06

**Authors:** Melda Tozluoglu, Yanlan Mao, Paul A. Bates, Erik Sahai

**Affiliations:** 1 MRC/UCL Laboratory for Molecular Cell Biology, University College London, London, UK; 2 Biomolecular Modelling Laboratory, London Research Institute, Cancer Research UK, London, UK; 3 Tumour Cell Biology Laboratory, London Research Institute, Cancer Research UK, London, UK

**Keywords:** cancer cell motility, plasticity of motility, adaptation to extracellular matrix, plasma membrane blebbing, cell–extracellular matrix adhesion feedback

## Abstract

Cells can move through extracellular environments with varying geometries and adhesive properties. Adaptation to these differences is achieved by switching between different modes of motility, including lamellipod-driven and blebbing motility. Further, cells can modulate their level of adhesion to the extracellular matrix (ECM) depending on both the level of force applied to the adhesions and cell intrinsic biochemical properties. We have constructed a computational model of cell motility to investigate how motile cells transition between extracellular environments with varying surface continuity, confinement and adhesion. Changes in migration strategy are an emergent property of cells as the ECM geometry and adhesion changes. The transition into confined environments with discontinuous ECM fibres is sufficient to induce shifts from lamellipod-based to blebbing motility, while changes in confinement alone within a continuous geometry are not. The geometry of the ECM facilitates plasticity, by inducing shifts where the cell has high marginal gain from a mode change, and conserving persistency where the cell can continue movement regardless of the motility mode. This regulation of cell motility is independent of global changes in cytoskeletal properties, but requires locally higher linkage between the actin network and the plasma membrane at the cell rear, and changes in internal cell pressure. In addition to matrix geometry, we consider how cells might transition between ECM of different adhesiveness. We find that this requires positive feedback between the forces cells apply on the adhesion points, and the strength of the cell–ECM adhesions on those sites. This positive feedback leads to the emergence of a small number of highly adhesive cores, similar to focal adhesions. While the range of ECM adhesion levels the cell can invade is expanded with this feedback mechanism; the velocities are lowered for conditions where the positive feedback is not vital. Thus, plasticity of cell motility sacrifices the benefits of specialization, for robustness.

## 1. Introduction

Motile cells are required to navigate through a variety of extracellular conditions, both in terms of the geometry and adhesiveness of the extracellular matrix (ECM). These changing environments have different motility requirements [1]. Certain ECM geometries favour a mode of motility that uses actin polymerization within lamellipodia to push the membrane forward. By contrast, other geometries favour migration dominated by high actomyosin contractility and hydrostatic pressure pushing the plasma membrane forward. The latter form of migration is characterized by membrane blebs. Similarly, different cell–ECM adhesion levels result in different motility modes becoming more efficient [2]. To maintain motility in response to changes in the extracellular environment, cells can adapt their behaviour. The plasticity of cell motility can be exploited in pathological contexts such as the spread of cancer cells through the body. However, it is unclear what regulatory mechanisms confer this adaptability and if this plasticity comes at the cost of the benefits of specialization. A large number of inter-connected physical and biochemical parameters involved in determining the efficiency of cell migration, many of which may also determine changes in migration strategy [3]. This complexity can be daunting; however computational modelling has recently started to provide analytic frameworks that enable the precise, and context-dependent role of individual phenomena to be determined.

Beyond biochemical perturbation of a selected motility driver, changing the ECM geometry can induce motility mode changes. Leucocytes can move in a lamellipod-dependent mode on *in vivo* surfaces, such as the endothelial lining, and switch to a low adhesion, flexible morphology mode of motility within interstitial collagen [4,5]. Adult skeletal muscle stem cells crawl on the basal lamina, and during penetration of the basal lamina and through the meshwork of myofibres, they switch to movement with a flexible morphology and plasma membrane blebbing [6]. The plastic nature of cell motility under ever-changing extracellular conditions is frequently observed, yet our understanding of the factors enabling these shifts is limited. A better understanding of these factors are essential, in both promoting cell movement, such as in stem cell treatments; and inhibiting it, such as targeting cancer cell motility during metastasis.

In the current work, we focus on how migrating cells adapt to changes in ECM geometry and adhesiveness. We build upon our previously reported computational model of cell motility that incorporates flexible cell morphology, plasma membrane blebbing, lamellipodia formation and interactions with the ECM filaments [2]. First, we show that shifts in modes of motility in response to changes in matrix geometry are an emergent property of the model. These changes are linked to the confinement-driven hydrostatic pressure changes of the cell and the availability of surfaces to spread lamellipodia. Within confined environments, changes in ECM adhesiveness can also lead to changes in migration mode. However, changes in cell–matrix adhesion on unconfined surfaces frequently lead to cell detachment and loss of migration. To overcome this difficulty, we investigate the influence of introducing a feedback between the strength of cell–ECM adhesions and the forces applied on junction points [7,8]. Incorporation of this feedback to the model is sufficient for formation of spatially discrete high-adhesion regions, reminiscent of focal adhesions. We show that cells equipped with mechanosensing and adhesion regulation have higher robustness when faced with changes in adhesion levels, but their velocities are lower than the peak velocities at optimum adhesion levels. Overall, the observed plasticity of cell motility ensures cells continue movement under changing conditions; and comes at the cost of peak velocities cells could reach, under conditions optimized for the current extracellular state.

## 2. Results

### 2.1. A two-phase solution to cell motility mode efficiency is mapped to distinct regions of cell–extracellular matrix adhesion and extracellular matrix geometry spectrums

To study the plasticity of cell motility, we use a physical model of cell dynamics [2] (electronic supplementary material, figure S1*a*). The model cell has flexible morphology; it can form two basic types of protrusions: lamellipodia and plasma membrane blebs. The ECM fibres are defined explicitly, allowing for investigation of detailed interactions between the ECM geometry, cell–ECM adhesion regulation and cell motility mechanisms (see ‘Cell motility model’ section in Material and methods). Our previous version of the model could reproduce many features of cell migration, however the considerations of membrane blebbing and cell–ECM adhesion were over-simplified. In this study, we improve these aspects of the model and increase its computational efficiency (see electronic supplementary material, Initiation of blebs on retracting blebs section and figure S1*b*–*d*). Following implementation of these changes, we use our model to investigate the regulatory mechanisms that enable plasticity of cell motility, under changing environment conditions.

**Figure 1.**
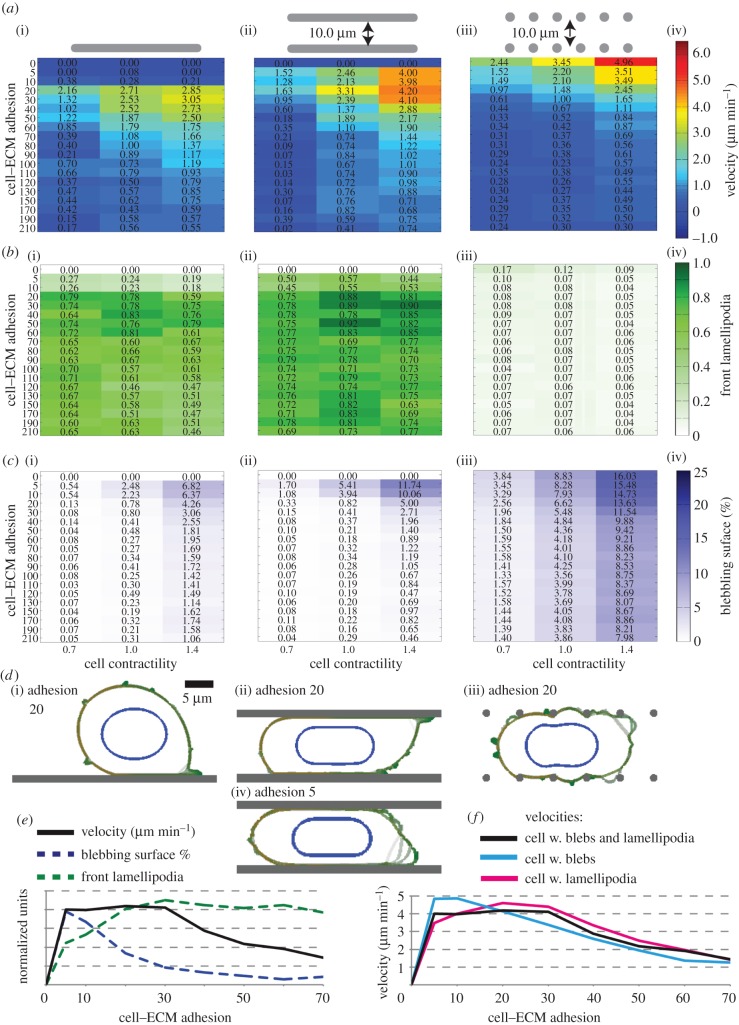
Motility mode characterization. (*a*) Velocity heatmaps showing the performance of a cell with blebs and lamellipodia (i) on a surface, (ii) in a confined continuous and (iii) in a confined discontinuous environment; *y*-axis: adhesion levels, *x*-axis: overall cell contractility. Cell polarity is 50% increased contractility at the cell rear accompanied by a 50% reduction of contractility at cell front, and 40% reduced ERM levels at cell front. (iv) Colourbar is valid for *a*(i–iii). (*b*) Front spreading lamellipodia score and (*c*) blebbing surface percentage heatmaps, organization same as in (*a*). (*d*) Simulation snapshots for contractility 1.4, (i) from *a*(i), adhesion 20, (ii) from *a*(ii), adhesion 20, (iii) from *a*(iii), adhesion 20, and (iv) from *a*(ii), adhesion 5. Scalebar in (i) is 5 µm, and valid for all (i–iv). (*e*) Plot of velocity (black), blebbing score (dashed blue) and front spreading lamellipodia score (dashed green), against cell–ECM adhesion strength (*x*-axis). The cell is in a confined continuous environment, with contractility set to 1.4. Data are extracted from heatmaps *a*(ii),*b*(ii),*c*(ii), and *y*-values are normalized to show the position of peaks. (*f*) Cell velocity (µm min^−1^) for a cell with only blebs (cyan), only lamellipodia (magenta) and both blebs and lamellipodia (black), against cell–ECM adhesion strength. The cells are within a confined continuous environment. Cell contractility is 1.4, data are extracted from *a*(ii), and electronic supplementary material, figure S2*a*(ii). The distinct peaks of bleb only and lamellipodia only motilities coincide with the larger plateau of the cell with both blebs and lamellipodia.

We first investigate the cell velocity performance under various ECM geometries, cell–ECM adhesion levels and actomyosin contractility levels. In accordance with experimental observations, the cells in simulations are polarized to have increased myosin and cortex–membrane linkage (ERM protein) at the cells' rear [9–12] (electronic supplementary material, figure S1*e*–*g*), as a means of generating robust directional motility. The lamellipodia formation rates are inversely linked to myosin concentrations on the cell surface, mimicking the antagonistic effect of Rho–Rac signalling [13]. Figure 1*a* shows that significant cell velocities can be achieved in all the matrix geometries tested. An intermediate level of cell–ECM adhesion is optimal on unconfined surfaces (figure 1*a*(i)). Lower levels of cell–ECM adhesion can be tolerated in confined continuous environments (figure 1*a*(ii)) and matrix adhesion is not required in confined discontinuous environments (figure 1*a*(iii)). Our simulations with polarized lamellipodia in the absence of myosin polarity suggest polarity in the contractile forces is necessary for consistent and rapid directional movement of the cell body (electronic supplementary material, figure S1*h*, also see ‘A detailed description of the cell motility model’ section). These results are consistent with our findings using the previous version of the model [2]. Therefore, our modifications to make the model more computationally efficient and incorporating blebs on blebs have not fundamentally altered the behaviour of the model.

To obtain a quantitative insight into cell migration mode changes, metrics are implemented for the extent of membrane blebbing and lamellipodia. The extent of plasma membrane blebbing is scored as the percentage of blebbing cell surface. The contribution of lamellipodia on cell motility is scored by the lamellipodia spreading on ECM surfaces. At each time point, the binary lamellipodia score is 1 if the cell has spreading lamellipodia in the selected direction (front or rear), and 0 otherwise (see electronic supplementary material, ‘Protrusion Scores’ section). These metrics are then calculated for all matrix adhesion and contractility conditions tested in figure 1*a*. This reveals lamellipodia are dominant when cells are moving on or between continuous surfaces (figure 1*b*), and blebs dominate in discontinuous environments (figure 1*c*). In line with experimental data, increasing contractility correlates with increased blebbing [14]. Exemplars of these migration modes for cells with a cell–ECM adhesion value of 20 and overall contractility of 1.4 are shown in figure 1*d*(i–iii). A further feature of this analysis is that reduced adhesion favours membrane blebbing (figure 1*d*(iv)). For a cell moving in a confined continuous environment with a contractility value of 1.4, the profile of cell velocity with varying cell–ECM adhesion suggests a single broad peak of velocity between ECM adhesion values of 5 and 30 (figure 1*a*(ii),*e*). However, the blebbing and lamellipodia profiles indicate this single broad velocity peak comprises two different migratory behaviours (figure 1*e*). Cells migrate with extensive blebs at low ECM adhesion. Above ECM adhesion of 20, there are very few blebs, and lamellipodia dominate (figure 1*d*(ii),(iv)). Further support for the relationship between matrix adhesion and the mode of migration is obtained from velocity–adhesion relationships of cells restricted to only blebbing or only lamellipodia-driven migration (electronic supplementary material, figure S2*a*–*c*). The maximal velocities for a blebbing only strategy are achieved with cell–ECM adhesion of 5–10, whereas the maximal velocities for lamellipodia-driven cells are 20–30 (figure 1*f*). Interestingly, the maximal velocities obtained when cells are restricted to a single migratory strategy are higher than when both blebbing and lamellipodia are permitted. This indicates that ability to employ different migration modes comes at some cost to the migrating cell's maximal velocity.

The analysis above demonstrates a two-phase solution to the problem of cell migration in different matrix geometries or with different ECM adhesion levels. The lamellipodia-driven migration phase dominates in the presence of continuous surfaces and higher matrix adhesion. The bleb-driven migration phase is observed in confined environments that are discontinuous, or with low cell–ECM adhesion. These data raise the possibility that cells may undergo transition between these two phases or migration modes, in response to encountering changes in either the geometry or adhesiveness of the ECM.

### 2.2. Plasticity in motility mode is an emergent property of the model

**Figure 2.**
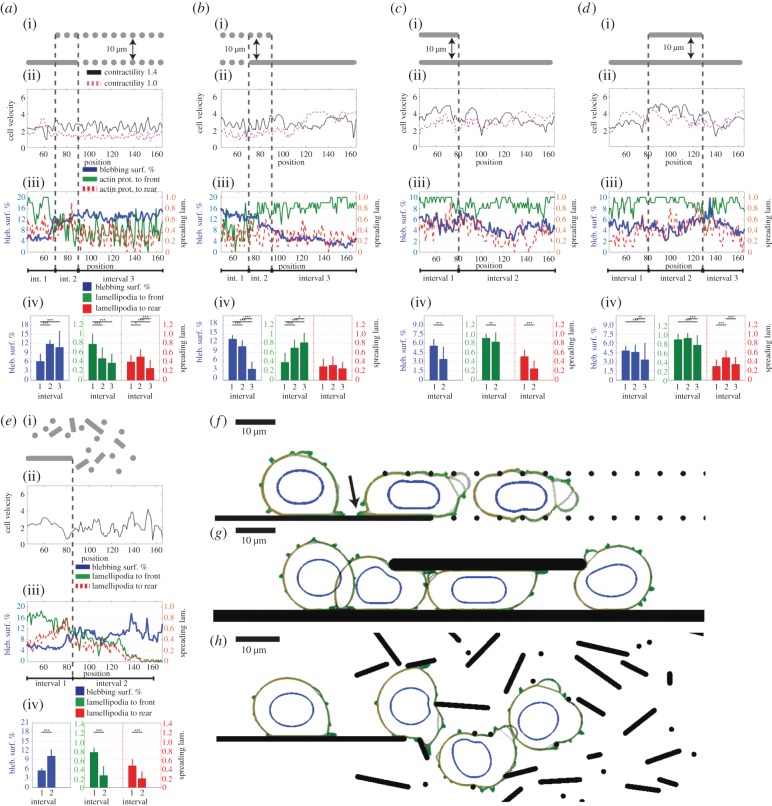
Cell behaviour in changing ECM geometry. (*a*–*e*) Cells can form plasma membrane blebs and lamellipodia. Overall contractility of the cell 1.4, 50% increased contractility at the cell rear accompanied by a 50% reduction of contractility at cell front. Forty per cent reduced ERM at cell front. Cell ECM adhesion is 20 units. Data points taken at every second of simulation time; binned at 1 µm intervals with respect to the position of cell centre. All plots averaged over at least 10 simulations. (i) Environment schematic, (ii) instantaneous cell velocity (µm min^−1^), calculated every minute in solid black, instantaneous velocities for cells mimicking a predominantly lamellipod-dependent cell type are plotted in dashed pink for ease of comparison, further details of this set-up are given in electronic supplementary material, figure S3*g*–*j*. (iii) Front spreading lamellipodia score (green), rear spreading lamellipodia score (dashed red) and percentage of blebbing cell surface (blue) plotted as a function of cell position within the environment. Note the scale changes in plots for blebbing scores in *c*,*d*. (iv) Statistical analysis of average protrusion scores for the position intervals marked on the environment schematic (i). Colour coding same as (ii). Two-tail *t*-test carried out between scores of each interval within the environment, *p < 0.05, **p < 0.01 and ***p < 0.001. (*a*) Cell in transition from an unconfined surface to a confined discontinuous environment; (*b*) from a confined discontinuous environment to an unconfined surface; (*c*) from a confined continuous environment to an unconfined surface; (*d*) from an unconfined surface to a confined continuous environment, and back to the unconfined surface; (*e*) from a surface to an *in vivo*-mimetic environment. (*f*) Snapshots overlaid from a representative simulation of (*a*). Arrow points at the lamellipod spreading towards the cell rear, within the transition zone. See electronic supplementary material, movie S1. (*g*) Model snapshots for (*d*), see electronic supplementary material, movie S2; (*h*) model snapshots for (*e*), see electronic supplementary material, movie S3. For (*f*–*h*), scale bars, 10 µm.

We hypothesized that cells within our model would dynamically alter their motility mode if the geometry of the matrix they encountered changed. Therefore, we simulate cells moving through changing environment geometries, as combinations of (i) unconfined surfaces, (ii) confined continuous environments, (iii) confined discontinuous regular matrices of fibres (figure 2*a*–*d*) and finally (iv) random meshes that are generated based on *in vivo* ECM geometries [2] (figure 2*e*). In these simulations, cells are permitted to use both lamellipodia and plasma membrane blebbing.

When the cells switch from moving on a surface into a discontinuous matrix (figure 2*a*(i)), they can maintain their movement rate (figure 2*a*(ii)—solid black line), yet there is a significant decrease in spreading lamellipodia both towards the cell front and the rear, and a significant increase in blebbing surface percentage (figure 2*a*(iii),(iv)). Moving from a surface to a discontinuous matrix pushes the cell towards a more blebbing motility mode. Thus, the model can recreate a transition between different migratory strategies in response to changing matrix geometries. These changes occur without necessitating any regulation at the ‘biochemical’ level, such as varying the probability of protrusion initiation, actomyosin contractility or cell–ECM adhesion. The regulation is carried out by the availability of resources at the physical level, first by the lack of continuous surfaces to stabilize lamellipodia, and second, by the increase in internal cell pressure due to cell shape change, driving increased blebbing [15] (electronic supplementary material, figure S3*a*). Although the A375 melanoma that we use for the parameterization of our model have a predominantly blebbing motility phenotype, simulations for a parameter set similar to the largely lamellipodia-dependent MDA-MB-231 breast cancer cells demonstrate the same transitions, albeit with higher protrusion scores on surfaces (figure 2*a*(ii)—dashed pink line; electronic supplementary material, figure S3*g*, see also ‘A detailed description of the cell motility model’ section). Thus, plasticity of the migration mode is a generic feature observed with parameters representing both MDA-MB-231 breast cancer and A375 melanoma cells.

During the transition (figure 2*a*(i)—interval 2), when the ECM surface comes to an end (cell centre at approx. 70 µm), the lamellipod spreading towards the front stops elongating and retracts, reducing the pulling forces on the cell rear. This allows for a transient increase in rearward lamellipodia score (figure 2*a*(iv)), which is reduced below that of the levels on a continuous surface, as the cell progresses through the discontinuous environment (figure 2*a*—interval 3). The percentage of the cell surface undergoing blebbing increases through the transition period and into the discontinuous matrix (figure 2*f*; electronic supplementary material, movie S1). Under confinement at the adhesion level of 20, the force balance between the ECM adhesion and contractility leads to higher curvature at the cell tips relative to the surface curvature of an unconfined cell. As formulated with Laplace's law, the higher curvature generates higher pressures under unchanged surface tension resulting from actomyosin forces. These local changes in the curvature lead to cells with higher internal pressure in confined environments and to more extensive blebbing (electronic supplementary material, figure S3*a*).

An inverse transition occurs while the cells are moving onto a surface emerging from a discontinuous matrix (figure 2*b*; electronic supplementary material, figure S3*b*,*h*). When the cell leaves the confined region, the relaxation of the cell shape leads to reduction in cell pressure, which reduces blebbing. As the lamellipodia are stabilized and spread the cell further, the curvature of the cell body is also reduced, leading to further reduction in internal cell pressure. The force balance between contractile and adhesive forces determines the extent of spread of the cell, hence the internal pressure and the concomitant blebbing extent (electronic supplementary material, figure S3*b*).

### 2.3. Continuous surfaces induce actin polymerization-based motility beyond optimal levels

Cells moving through transitions between surfaces and confined continuous environments show less pronounced changes in their motility modes. The cells stay predominantly in the lamellipod-based phenotype at all times (figure 2*c*,*d*).

Through the transitions between unconfined and confined regions, the lamellipodia scores stay above 0.75 at all times, indicative of cells stabilizing forward lamellipodia on the surfaces, and maintaining them through the environment changes. Inside the confined regions, the blebbing score is relatively higher, yet still below 6%, and the front spreading lamellipodia score is above 0.9. This suggests the cell stays in a lamellipod-dominated phenotype through the transitions between confined and unconfined surfaces (figure 2*c*,*d*,*g*; electronic supplementary material, movie S2 and figure S3*i*,*j*).

Lamellipodia-driven motility is already more effective than blebbing motility on both unconfined surfaces and under confinement, at the adhesion level of 20 (electronic supplementary material, figure S2*a*(i,ii)). At this adhesion, we cannot distinguish whether the cell selectively stays in the optimum motility mode, or the cells are able to change into blebbing motility only when the lamellipodia are forced to retract by the environment. To clarify this point, we ran simulations at the cell–ECM adhesion level of 10, where blebbing motility is more effective within confined continuous geometries (blebbing only velocity—4.9 µm min^−1^ versus lamellipod-only velocity—4.0 µm min^−1^; electronic supplementary material, figure S2*a*(ii)). Here, the cells start having higher blebbing scores, yet, the front spreading lamellipodia scores still stay at 0.8 or higher under confined regions (electronic supplementary material, figure S3*c*,*d*). This indicates there is not a complete shift in the cell motility mode, even under conditions where a blebbing motility mode would have been more efficient.

Although the motility mode is not shifted, the increase observed in blebbing is related to the pressure changes of the cell. Under high cell–ECM adhesion, spreading lamellipodia are stabilized more successfully, and the cell rear is harder to detach. Then the cell spreads more, reducing the internal cell pressure, therefore suppressing blebbing (electronic supplementary material, figure S3*e*,*f*, for adhesion 20, compared to figure S3*c*(iv),*d*(iv) for adhesion 10). Shape changes induced by confinement and low adhesion can lead to the generation of higher intracellular pressures with a concomitant increase in blebbing.

When the cells transition into *in vivo*-mimetic environments from unconfined surfaces, they change into a more blebbing-dominated motility, with significant reduction in lamellipodia scores and an increase in blebbing (figure 2*e*,*h*; electronic supplementary material, movie S3). The characteristics of this transition are similar to those observed in cells moving from unconfined surfaces into confined discontinuous environments (figure 2*a*). Taken together, these data demonstrate that changes in matrix geometry lead to changes in the mode of cell migration. Further, these changes result in the utilization of a cell migration mode that is well suited, although not necessarily optimal, for the matrix geometry. Importantly, these changes occur without any global changes in the overall actin protrusion probability, actomyosin contractility, cell–matrix adhesion or other biochemical properties of the cells. Rather, they emerge as a natural consequence of the geometry of the environment that stabilizes/destabilizes lamellipodia and influence cell pressure.

### 2.4. Cell polarity control mechanisms are required for plasticity

**Figure 3.**
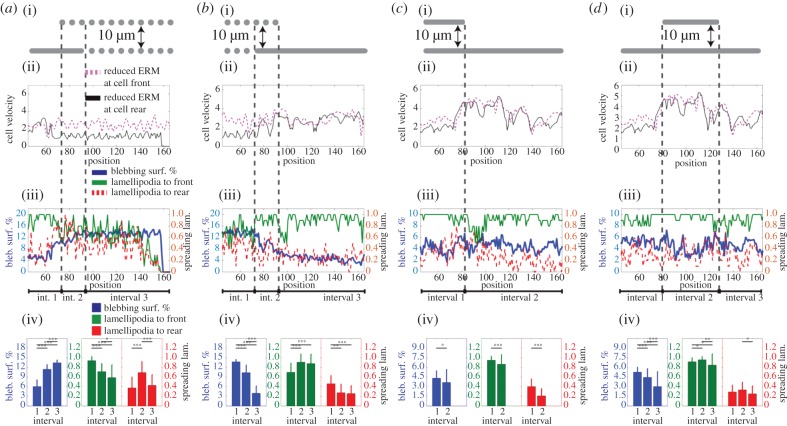
The effect of cortex–membrane adhesion polarity on motility mode shifts. All cellular conditions are the same as figure 2, except for localization of cortex–membrane adhesion polarity. Cells have 40% reduced ERM levels at the cell rear. Data binning and representation are the same as in figure 2. In panel (ii) of (*a*–*d*), the instantaneous velocity profiles of the cells with higher blebbing at the cell rear are plotted in black. Corresponding co-localized ERM and myosin polarity scenarios, with increased blebbing at the cell front, are plotted in dashed pink, for ease of comparison (repetitive data from figure 2). See electronic supplementary material, movie S4.

Up to this point, we have analysed cell behaviour with co-localized myosin and cortex–membrane linkage (ERM protein) polarity, where both myosin and ERM are higher at the cell rear. This was based on experimental evidence of co-localization of the myosin and ERM proteins at the rear of migrating cells [9–11] (electronic supplementary material, figure S1*e*,*f*); however, these two parameters do not necessarily need to be similarly polarized in all physiological settings. To investigate the roles of the relative polarity of these proteins on plasticity of cell motility, we investigate cell behaviour with myosin and ERM proteins localizing at opposite ends of the cell, where myosin is higher at the cell rear and ERM is higher at front. This leads to the cells having a higher probability of blebbing at the rear. The change in polarity does not change the motility mode shifts with transitions of ECM geometry (figure 3), but we see an approximate 50% drop in instantaneous velocity profiles when the cell successfully shifts into a blebbing mode of motility (figure 3*a*(ii),*b*(ii); electronic supplementary material, movie S4(i)). With the blebs forming at the cell rear and competing with the contractile cortex, they cause a velocity reduction, rather than facilitating forward movement. The velocity is indeed reduced to a level below that of a cell without the ability to form blebs (electronic supplementary material, figure S2*a*(iii)). Motility in confined continuous environments is not adversely affected due to the dominant lamellipodia (figure 3*c*,*d*; electronic supplementary material, movie S4ii). This behaviour is reproducible at higher cell polarities (electronic supplementary material, figure S4). However, we previously noted that this organization of actomyosin and cortex–plasma membrane linkage polarities yielded the highest velocities on non-confined surfaces [2]. Therefore, although adaptation of migratory strategy is facilitated by similar polarization of the contractile actomyosin network and cortex–plasma membrane linkers at the cell rear, it is not optimal for migration on a two-dimensional surface.

### 2.5. Varying cell–extracellular matrix adhesion challenges plasticity

**Figure 4.**
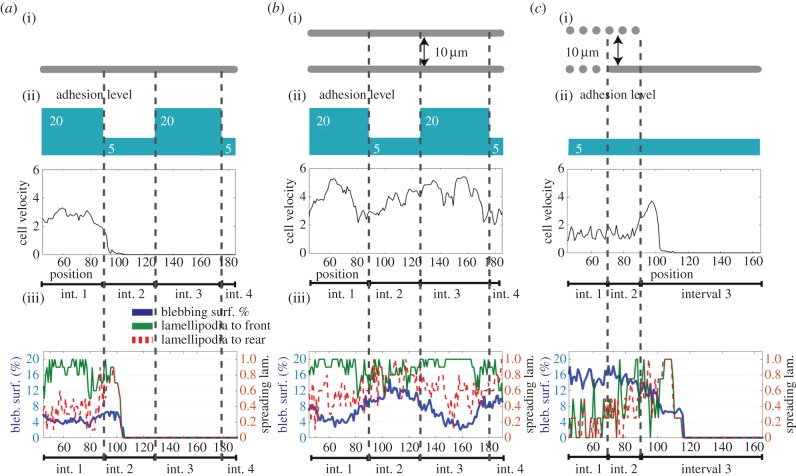
Impact of cell–ECM adhesion on plasticity of motility. (*a*,*b*) Cell motility in environments with changing adhesion levels. Data binning and all cellular conditions are the same as figure 2, except for adhesion levels, which are indicated in panels (ii). (i) Environment schematic, (ii) adhesion profile schematic and instantaneous cell velocity and (iii) lamellipodia and blebbing scores. (*a*) Unconfined surface. (*b*) Confined continuous environment. (*c*) Environment transition from a confined continuous environment to an unconfined surface, at cell–ECM adhesion level of 5 units.

In the simulations carried out above, the adhesiveness of the ECM has been constant; however, cells moving through tissue will encounter matrices of different composition that will have different adhesive characteristics. Therefore, we investigate the cell behaviour with changing adhesion conditions. We simulate a cell moving on a surface that changes its adhesiveness between 20 and 5 (figure 4*a*). Under these conditions, the cell cannot pass the low adhesive region and is detached. This indicates that variations in the adhesion properties of the ECM hinder a cell's ability to move continuously on a surface. However, under the same conditions within confinement, the cells rapidly change between motility modes as the cell–ECM adhesion changes (figure 4*b*). When the cell contacts more adhesive ECM (value 20), lamellipod-driven motility dominates; blebs become dominant when ECM adhesiveness is 5 [16]. The analysis in figure 4*a*,*b* predict that a cell will be unable to transition successfully from a confined environment to an unconfined environment if the cell–ECM adhesiveness is only 5. We confirm this prediction in figure 4*c*. Together, these simulations demonstrate that changes in matrix adhesiveness can induce changes in migration strategy and that the ability to effectively maintain cell migration only occurs within a narrow range of cell–ECM adhesion values. To enable plasticity across a wider range of matrix adhesiveness, a mechanism to modulate cell–ECM adhesion would be beneficial.

### 2.6. A simple feedback between tension and cell–ECM adhesion strength is sufficient for formation of focal adhesion-like structures

**Figure 5.**
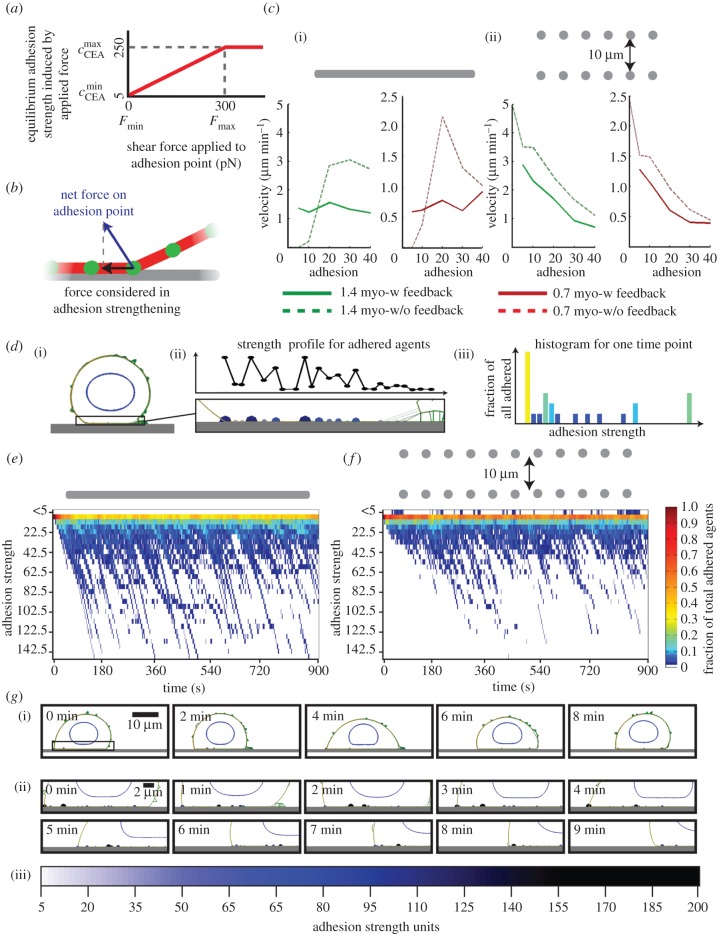
Implications of positive feedback between cell–ECM adhesion strength and tension on adhesion sites. (*a*) Representation of tension–adhesion feedback curve. The equilibrium adhesion concentration is induced by shear force on the adhesion point. For cell performance with different parameter sets, see electronic supplementary material, figure S5. (*b*) Schematic of the forces included in tension–adhesion feedback mechanism. (*c*) Cell velocity as a function of adhesion on (i) an unconfined surface, (ii) confined discontinuous environment, with (solid lines) and without (dashed lines) tension–adhesion feedback. For the simulations with mechanosensing, initial adhesion concentration 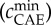 and the lower limit of the force magnitude to start strengthening (*F*_min_) are shifted on the tension–adhesion feedback curve (*a*). Data for overall contractility 0.7 are in green and 1.4 in red. Cells can form plasma membrane blebs and lamellipodia; polarity is the same as in figure 2. (*d*) Adhesion heatmap generation methodology. (i) Model snapshot for a selected time point. (ii) Close up of adhered agents in (i), and the adhesion profile plot for the adhered agents only. (iii) Adhesion strength histogram for adhered agents, binned at 5 unit intervals. The histogram corresponds to the snapshot in (i) and (ii). Bars are colour coded with respect to fraction magnitude, which also reflects the representation in heatmaps in (*e*,*f*). (*e*,*f*) Heatmaps for adhesion concentration of adhered agents, binned at 5-unit intervals. These are the colour-coded histograms as in (*d*(iii)), stacked in time horizontally. The *y*-axis indicates the adhesion level bins, and *x*-axis the simulation time (s). The cell has a minimum adhesion concentration of 5 units, polarity same as in (*c*), overall contractility level is 1.4. (*e*) Data on unconfined surface and (*f*) within a confined discontinuous environment. Colourbar valid for (*e*,*f*). A small number of adhesion points reach very high levels, while majority stays within the initial bin of 5–10 units. (*g*) Simulation snapshots showing the adhesion strengthening on a small number of nodes in a neighbourhood, (*g*(ii)) has close shots of the boxed region in (*g*(i)). (iii) Colourbar for the adhesion strength of cell surface agents in snapshots (i) and (ii).

Experimental studies have extensively documented the ability of integrin-mediated adhesions to become stronger when the forces exerted upon them increase [7,8,17]. Briefly, the application of force to adhesion complex proteins such as p130Cas and talin causes changes in protein folding [18–20]. This in turn alters the availability of surfaces for biochemical regulation, either at the level of phosphorylation or protein–protein interaction, leading to stronger cell–matrix adhesions. We propose that a regulatory mechanism for the cell–ECM adhesion in response to applied forces is a good candidate to increase the plasticity of cell motility. We implement a tension–adhesion feedback in our model and test its effects on cell motility. In this model, the forces acting on a local adhesion point can induce changes in the adhesion strength at that point. This is modelled similar to an ‘equilibrium concentration’ (figure 5*a*): the forces acting on the adhesion determine the target adhesion strength, and the ‘concentration’ of the adhesion point is modulated towards the equilibrium with a turnover time comparable to actin turnover (see electronic supplementary material, ‘Cell–ECM adhesion strengthening’ section). Within this framework, the characteristics of the feedback mechanism are determined by the adhesion ranges available for the cell to modulate, and force ranges it can respond to. The low end of the adhesion range defines the initial adhesion strength immediately at formation. The high end of the adhesion range is the saturation point, where further increase in the tension applied to the adhesion point will not induce additional strengthening. The lower end of the force range identifies the minimal force magnitude that the cells can sense, and the high end of the force range corresponds to the force level that induces adhesion saturation (figure 5*a*).

To characterize the influence of the feedback mechanism, we test a series of ranges for both the adhesion and force components, and whether adhesion strengthening responds to forces exerted parallel or perpendicular to the plane of the ECM (electronic supplementary material, figure S5). This analysis reveals that the improvements in cell velocity at low adhesions on unconfined surfaces mostly come at the cost of reduction in cell velocity under other conditions. We show that the feedback response is most effective when the forces acting on the plane of the ECM fibre facilitate strengthening (figure 5*b*,*c*(i); electronic supplementary material, figure S5), the adhesion range is 5–250 units, and any positive force induces strengthening, with saturation at 300 pN (figure 5*a*,*b*).

The feedback mechanism does not cause a uniform increase in adhesion across all points of the cell in contact with the ECM. Accompanying a limited increase in overall adhesion strengths, a small number of strong adhesions emerge. The overall adhesion profiles are demonstrated as adhesion strength heatmaps. These show the distribution of adhesion strengths in time. In each simulation, we take the adhesion strength distribution of the adhered nodes every second (figure 5*d*(i),(ii)). For each time point, we bin the adhesion levels at 5-unit intervals and prepare a histogram of adhesion strengths. Then the histograms are colour coded for the fraction values (figure 5*d*(iii)). Then different time points are stacked horizontally in the form of heatmaps, with the colour coding from the histograms (figure 5*e*,*f*). The heatmaps demonstrate the majority of adhesions stay at low levels, with a very small fraction of adhesion points continuing on to form strong adhesion sites, resembling focal adhesion saturation. The frequent downward diagonal patterns are typically the result of the strengthening of a single adhesion over time. They terminate when the adhesion becomes detached.

The strong adhesions form as a result of competitive strengthening between nearby adhesion points. When one of two neighbouring—equally strong—adhesions detaches stochastically, the force on the intact adhesion increases, causing a further strengthening. With a series of such successive selection events, a small number of strong adhesions emerge (figure 5*g*). Under same conditions, if the perpendicular components of the pulling forces are facilitated in adhesion strengthening as opposed to shear forces, the cells can increase the adhesions at a smaller range (electronic supplementary material, figure S6*a*), perpendicular strengthening is not effective at generating strong adhesions in the front half of the cell. The profiles emerging from shear forces inducing adhesion strengthening are closer to the experimentally observed patterns (electronic supplementary material, figure S6*b*). This is in line with the cell geometry-dependent changes on the forces the cells can apply on the substrate, and concomitant changes in focal adhesion maturation at progressing stages of cell spreading [21].

### 2.7. Adhesion feedback improves plasticity of cell motility at the cost of peak velocities

**Figure 6.**
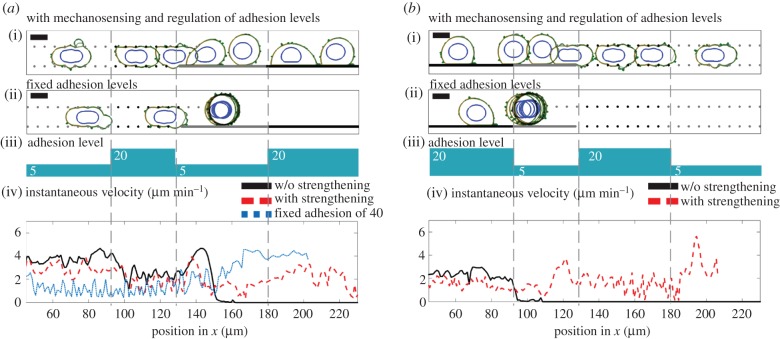
Influence of mechanosensing in cell motility plasticity, upon changes in the ECM geometry and adhesion. Panels (i) overlaid snapshots from simulation with mechanosensing (positive feedback between tension applied on adhesion points and cell–ECM adhesion). Snapshots are taken at 15-min intervals. (ii) Same as (i), for a cell without mechanosensing. Fixed adhesion levels are induced by the ECM. (iii) The schematic showing the adhesion level induced by the ECM, this determines 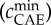 and (*F*_min_) for (i) and the fixed adhesion level for (ii). (iv) Instantaneous velocity plots measured every minute and binned at 1 µm intervals with respect to the position of the cell centre. Simulations with mechanosensing are in dashed red, and simulations of fixed adhesion are plotted with solid black lines. In (*a*), instantaneous for (i) and the fixed adhesion level for (ii) velocity for fixed adhesion level of 40 within the same environment geometry is plotted in dashed blue, to compare the adhesion feedback strengthening effect with constitutively high adhesion. (*a*) Environment geometry is changing from confined discontinuous to unconfined continuous (see electronic supplementary material, movie S5). (*b*) Environment geometry is changing from unconfined surface to confined discontinuous. For all the simulations, cells have an overall contractility of 1.4, 50% increased contractility at the cell rear accompanied by a 50% reduction of contractility and 40% reduced ERM levels at the cell front. Data are an average of at least 10 simulations.

Finally, we investigate cell behaviour upon encountering changes in adhesion strength as well as ECM geometry (figure 6). To simulate changing ligand content on ECM surface, we change the basal adhesion value in the absence of applied force (figure 5*a*). At low ECM ligand regions, the initial adhesions are formed at a minimum strength of 5 and are upregulated with every positive force. At high ligand regions, the adhesion points are initiated at the minimum strength of 20 and are upregulated only by forces above the corresponding force level (see electronic supplementary material, ‘Cell–ECM adhesion strengthening’ section). The cells that do not have the ability to regulate their adhesions have fixed adhesions of 5 units at low adhesion, and 20 units at high-adhesion regions.

Cells that regulate their adhesions move slower than the cells without the feedback mechanism in confined regions and unconfined surfaces with cell–ECM values of 20 (figure 6*a*-interval1/2, 6*b*–interval1; electronic supplementary material, movies S5 and S6). However, cells without feedback fail to move on unconfined surface regions with low ECM adhesion, while cells with the feedback can continue onwards. The same behaviour emerges when the cells are exposed to the changing adhesion levels at different orders (electronic supplementary material, figure S6*c*,*d*). Similar to simulations mimicking predominantly blebbing-dependent A375 melanoma cells, the simulations mimicking predominantly lamellipodia-dependent MDA-MB-231 breast cancer cells with mechanosensing ability display continuance of movement under changing adhesion conditions (electronic supplementary material, figure S6*c*(v)). In parallel with efficiency differences under changing adhesion levels, the influence of tension–adhesion feedback differs from that of a constitutively high-adhesion state in its increased performance within discontinuous environments and reduced performance on unconfined surfaces (figure 6*a*(iv)—dashed blue). The tension–adhesion feedback mechanism improves the robustness of cell motility when faced with rapid changes in ECM adhesiveness, however this comes at the cost of peak cell velocities. To conclude, the mechanisms that we identify to enable plasticity, including ability to use blebs and lamellipodia, co-localization of actomyosin and actin-membrane linkage at the rear, and adhesion strengthening all reduce maximal cell velocities, compared with the global optimum in each separate condition.

## 3. Discussion

The transition of cells between different migration modes in response to varying environmental changes is an important problem in health and disease. In our previous work, using a unified model that incorporates lamellipodia, blebbing and cell–ECM interactions, we identified different ECM geometries will have different cellular requirements and efficacy of invasion through a given matrix geometry will strongly depend on the motility mode [2]. In this study, building upon our model, we investigate the regulatory mechanisms that underlie transition in cell migration strategy. We construct a model that can maintain cell migration when challenged with changes in both matrix geometry and matrix adhesiveness. Strikingly, maintenance of cell movement through varying environments is associated with changes in migratory strategy. This is in agreement with experimentally observed plasticity in modes of cell migration [2,3,6,16,22–25].

We find a clear distinction between the performances of blebbing and lamellipodia-based motility modes on unconfined surfaces and confined discontinuous environments. Under all tested conditions, lamellipodia-based movement is more effective on a two-dimensional surface, and blebbing motility is more effective in confined discontinuous environments (electronic supplementary material, figure S2*a*(i),(iii)). In line with this clear-cut difference in performance, ECM geometry changes between unconfined continuous and confined discontinuous are sufficient to induce motility mode shifts to the cell, between dominant lamellipodia and blebbing (figure 2*a*,*b*). The observed transitions are consistent between cell types of different propensity for blebbing- and lamellipodia-based motilities (electronic supplementary material, figure S3*g*,*j*), with the cell type that is better equipped for a lamellipodia-based motility demonstrating higher protrusions scores as expected. The changes in mode of cell migration occur with no global changes in key actin and cell adhesion parameters, thus we propose that the switching of migration mode emerges from a combination of physical and localized feedback between cytoskeletal properties and the ECM. The change to bleb-driven motility in discontinuous confined environments is facilitated by the lack of surfaces to stabilize lamellipodia and increased intracellular pressure of the cell due to changes in cell geometry. Similarly, when ECM geometry changes from unconfined surfaces to the complex *in vivo* mimetic environment of interstitial collagen, the cell shifts into blebbing motility, albeit with occasional lamellipodia engaging continuous segments of matrix (figure 2*e*; electronic supplementary material, movie S3). In both examples, the confined environments are associated with increased intracellular pressure and this leads to increased membrane blebbing. The increase in pressure does not result from ‘biochemical’ changes in overall levels of actomyosin contractility. Instead, we believe that confinement in three-dimensional environments leads to higher levels of membrane and cell cortex curvature and, following basic physical principles, pressure and increasing curvature are positively correlated if cortical tension is constant. Thus, the most parsimonious explanation of the switch to blebbing migration needs only simple physics and not biochemical mechanisms.

Changes between unconfined and confined continuous surfaces have less pronounced influences on the cell motility mode. Confinement alone within continuous surfaces encourages higher blebbing (figure 2*c*,*d*), yet is not necessarily sufficient to enable the cell to convert from a lamellipodia-based to a blebbing motility mode, even under conditions where bleb-driven motility would be more beneficial (electronic supplementary material, figure S3*c*,*d*).

Increased intracellular pressure and blebbing *per se* are not sufficient to ensure effective migration in discontinuous environments. If myosin and ERM proteins localize at opposite ends of the cell, then the cell velocity decreases when the environment induces increased blebbing. Thus, for increased efficiency, the physical regulation from the ECM geometry additionally needs coordinated polarization of actomyosin contractility and linkage of the actin cortex to the plasma membrane. Indeed, such coordination is frequently observed in experimental systems [9,26] (electronic supplementary material, figure S1*e*,*f*).

The adhesion strength between the cell and the ECM is not solely under the control of the cell. Just as the cells encounter varying ECM geometries, they also encounter regions of varying adhesion ligand levels on ECM fibres. We therefore model transitions between matrices of varying adhesiveness. Migration into regions of low cell–ECM adhesiveness in confined environments is associated with increased membrane blebbing. This is in agreement with experimental observations. However, we observe that the cells’ ability to maintain migration in unconfined matrix environments is sensitive to the cell–ECM adhesion level. Specifically, if adhesion is below 10, then cells simply detach whenever they encounter an unconfined environment.

We explored whether biomechanical regulation with a positive feedback from the forces applied on the adhesion would enable cells to maintain migration even in unconfined matrices with low cell–ECM adhesion [8]. Our model enables us to explore properties of this feedback mechanism, which cannot be tested experimentally. In particular, we determine that strengthening of adhesion in response to forces parallel to the matrix leads to the most effective restoration of migration on unconfined surfaces with low cell–ECM adhesion. Strikingly, when this positive feedback mechanism is introduced into our model, our simulations suggest a small number of strong adhesions will emerge. Under conditions with changing ECM adhesiveness, the cells harbouring the positive tension–adhesion feedback perform more robustly than cells with fixed adhesion levels. These cells manage to continue movement in spite of the geometry and adhesion changes. They succeed through conditions where cells without adhesion regulation fail; albeit with lower velocities in some regions. Our model suggest that the effective regulation of cell–ECM adhesions will have a profound influence on the adaptability of the cell to changing environments, which indeed is observed in the motility regulation and plasticity of metastasizing breast cancer cells [27].

Our simulations suggest the changes in both the geometry of the ECM, and its adhesion ligand concentration, enable the cell to adapt its motility mode for persistent movement. Overall, the ability to cope with changes in the ECM content comes at the cost of high velocities, cells specialized for a given environment could reach. Plasticity of cell motility sacrifices the benefits of specialization, for robustness.

## 4. Material and methods

### 4.1. Cell motility model

Computational modelling has extensively been used to probe cell motility, from single protrusion dynamics, to interactions with and the structure of the ECM [28–43]. A detailed analysis of where our model fits within the available literature and its distinctive methodologies can be found in electronic supplementary material, ‘A detailed description of the cell motility model’ section.

Our model of single cell motility defines flexible cell morphology with the actomyosin cortex of the cell surface, the plasma membrane, local concentrations of proteins residing on the cell surface, a viscoelastic cell interior and an explicitly defined nucleus that can change shape (electronic supplementary material, figure S1*a*). This model structure allows for complete heterogeneity of contractility (myosin concentration), actin cortex density, cell cortex–plasma membrane adhesion (ERM protein concentrations) and cell–ECM adhesion over the cell surface.

Further details of the modelling methodology and parameters, the features we have added into our model for the current manuscript, the protrusion score calculation methods and the cell–ECM adhesion feedback formulation can be found in the electronic supplementary material.

## Supplementary Material

Supplementary Information

## Supplementary Material

Supplementary Figures

## References

[1] Charras G Sahai E 2014 Physical influences of the extracellular environment on cell migration. Nat. Rev. Mol. Cell Biol. 15, 813–824. (10.1038/nrm3897) 25355506

[2] Tozluoglu M Tournier AL Jenkins RP Hooper S Bates PA Sahai E 2013 Matrix geometry determines optimal cancer cell migration strategy and modulates response to interventions. Nat. Cell Biol. 15, 751–762. (10.1038/ncb2775) 23792690

[3] Friedl P Wolf K 2010 Plasticity of cell migration: a multiscale tuning model. J. Cell Biol. 188, 11–19. (10.1083/jcb.200909003) 19951899 PMC2812848

[4] Lämmermann T Germain R 2014 The multiple faces of leukocyte interstitial migration. Semin. Immunopathol. 36, 227–251. (10.1007/s00281-014-0418-8) 24573488 PMC4118216

[5] Madsen CD Sahai E 2010 Cancer dissemination: lessons from leukocytes. Dev. Cell 19, 13–26. (10.1016/j.devcel.2010.06.013) 20643347

[6] Otto A Collins-Hooper H Patel A Dash PR Patel K 2011 Adult skeletal muscle stem cell migration is mediated by a blebbing/amoeboid mechanism. Rejuvenation Res. 14, 249–260. (10.1089/rej.2010.1151) 21453013

[7] Choquet D Felsenfeld DP Sheetz MP 1997 Extracellular matrix rigidity causes strengthening of integrin–cytoskeleton linkages. Cell 88, 39–48. (10.1016/S0092-8674(00)81856-5) 9019403

[8] Galbraith C Yamada K Sheetz M 2002 The relationship between force and focal complex development. J. Cell Biol. 159, 695–705. (10.1083/jcb.200204153) 12446745 PMC2173098

[9] Lorentzen A Bamber J Sadok A Elson-Schwab I Marshall CJ 2011 An ezrin-rich, rigid uropod-like structure directs movement of amoeboid blebbing cells. J. Cell Sci. 124, 1256–1267. (10.1242/jcs.074849) 21444753

[10] del Pozo MA Nieto M Serrador JM Sancho D Vicente-Manzanares M Martínez-A C Sànchez-Madrid F 1998 The two poles of the lymphocyte: specialized cell compartments for migration and recruitment. Cell Adhes. Commun. 6, 125–133. (10.3109/15419069809004468) 9823463

[11] Soriano S Hons M Schumann K Kumar V Dennier TJ Lyck R Sixt M Stein JV 2011 *In vivo* analysis of uropod function during physiological T cell trafficking. J. Immunol. 187, 2356–2364. (10.4049/jimmunol.1100935) 21795598

[12] Asokan S Johnson HE Rahman A King SJ Rotty JD Lebedeva IP Haugh JM Bear JE 2015 Mesenchymal chemotaxis requires selective inactivation of myosin II at the leading edge via a noncanonical PLC*γ*/PKC*α* pathway. Dev. Cell 31, 747–760. (10.1016/j.devcel.2014.10.024) PMC4276478 25482883

[13] Sanz-Moreno V Gadea G Ahn J Paterson H Marra P Pinner S Sahai E Marshall CJ 2008 Rac activation and inactivation control plasticity of tumor cell movement. Cell 135, 510–523. (10.1016/j.cell.2008.09.043) 18984162

[14] Croft DR 2004 Conditional ROCK activation *in vivo* induces tumor cell dissemination and angiogenesis. Cancer Res. 64, 8994–9001. (10.1158/0008-5472.CAN-04-2052) 15604264

[15] Charras G 2005 Non-equilibration of hydrostatic pressure in blebbing cells. Nature 435, 365–369. (10.1038/nature03550) 15902261 PMC1564437

[16] Bergert M Chandradoss SD Desai RA Paluch E 2012 Cell mechanics control rapid transitions between blebs and lamellipodia during migration. Proc. Natl Acad. Sci. USA 109, 14 434–14 439. (10.1073/pnas.1207968109) PMC3437886 22786929

[17] Oakes PW Gardel ML 2014 Stressing the limits of focal adhesion mechanosensitivity. Curr. Opin. Cell Biol. 30C, 68–73. (10.1016/j.ceb.2014.06.003) PMC4459577 24998185

[18] Sawada Y Tamada M Dubin-Thaler BJ Cherniavskaya O Sakai R Tanaka S Sheetz MP 2006 Force sensing by mechanical extension of the Src family kinase substrate p130Cas. Cell 127, 1015–1026. (10.1016/j.cell.2006.09.044) 17129785 PMC2746973

[19] Yao M 2014 Mechanical activation of vinculin binding to talin locks talin in an unfolded conformation. Sci. Rep. 4, 4610 (10.1038/srep04610) 24714394 PMC3980218

[20] del Rio A Perez-Jimenez R Liu R Roca-Cusachs P Fernandez JM Sheetz MP 2009 Stretching single talin rod molecules activates vinculin binding. Science 323, 638–641. (10.1126/science.1162912) 19179532 PMC9339221

[21] Fouchard J Bimbard C Bufi N Durand-Smet P Proag A Richert A Cardoso O Asnacios A 2014 Three-dimensional cell body shape dictates the onset of traction force generation and growth of focal adhesions. Proc. Natl Acad. Sci. 111, 13 075–13 080. (10.1073/pnas.1411785111) PMC4246942 25157134

[22] Lämmermann T Sixt M 2009 Mechanical modes of ‘amoeboid’ cell migration. Curr. Opin. Cell Biol. 21, 636–644. (10.1016/j.ceb.2009.05.003) 19523798

[23] Starborg T 2008 Extracellular matrix and cell junctions: electron microscopy of collagen fibril structure *in vitro* and *in vivo* including three-dimensional reconstruction. In Introduction to electron microscopy for biologists (ed. Allen T), pp. 319–345. New York, NY: Elsevier. 10.1016/S0091-679X(08)00417-2 18617041

[24] Tyson RA Zatulovskiy E Kay RR Bretschneider T 2014 How blebs and pseudopods cooperate during chemotaxis. Proc. Natl Acad. Sci. USA 111, 11 703–11 708. (10.1073/pnas.1322291111) PMC4136620 25074921

[25] Zatulovskiy E Tyson R Bretschneider T Kay RR 2014 Bleb-driven chemotaxis of *Dictyostelium* cells. J. Cell Biol. 204, 1027–1044. (10.1083/jcb.201306147) 24616222 PMC3998804

[26] Madsen C 2015 STRIPAK components determine mode of cancer cell migration and metastasis. Nat. Cell Biol. 17, 68–80. (10.1038/ncb3083) 25531779 PMC5354264

[27] Deakin N Turner C 2011 Distinct roles for paxillin and Hic-5 in regulating breast cancer cell morphology, invasion, and metastasis. Mol. Biol. Cell 22, 327–341. (10.1091/mbc.E10-09-0790) 21148292 PMC3031464

[28] Strychalski W Guy R 2013 A computational model of bleb formation. Math. Med. Biol. 30, 115–130. (10.1093/imammb/dqr030) 22294562 PMC4104658

[29] Woolley T Gaffney EA Oliver JM Baker RE Waters SL Goriely A 2014 Cellular blebs: pressure-driven, axisymmetric, membrane protrusions. Biomech. Model. Mechanobiol. 13, 463–476. (10.1007/s10237-013-0509-9) 23857038

[30] Woolley T Gaffney EA Waters SL Oliver JM Baker RE Goriely A 2014 Three mechanical models for blebbing and multi-blebbing. IMA J. Appl. Math. 79, 636–660. (10.1093/imamat/hxu028)

[31] Charras G Coughlin M Mitchison TJ Mahadevan L 2008 Life and times of a cellular bleb. Biophys. J. 94, 1836–1853. (10.1529/biophysj.107.113605) 17921219 PMC2242777

[32] Tinevez J-Y Schulze U Salbreux G Roensch J Joanny J-F Paluch E 2009 Role of cortical tension in bleb growth. Proc. Natl Acad. Sci. USA 106, 18 581–18 586. (10.1073/pnas.0903353106) 19846787 PMC2765453

[33] Mogilner A Rubinstein B 2005 The physics of filopodial protrusion. Biophys. J. 89, 782–795. (10.1529/biophysj.104.056515) 15879474 PMC1366629

[34] Schaus T Borisy G 2008 Performance of a population of independent filaments in lamellipodial protrusion. Biophys. J. 95, 1393–1411. (10.1529/biophysj.107.125005) 18390606 PMC2479583

[35] Novak I Slepchenko B Mogilner A 2008 Quantitative analysis of G-actin transport in motile cells. Biophys. J. 95, 1627–1638. (10.1529/biophysj.108.130096) 18502800 PMC2483760

[36] Schlüter D Ramis-Conde I Chaplain M 2012 Computational modeling of single-cell migration: the leading role of extracellular matrix fibers. Biophys. J. 103, 1141–1151. (10.1016/j.bpj.2012.07.048) 22995486 PMC3446673

[37] Zaman M Kamm RD Matsudaira P Lauffenburger DA 2005 Computational model for cell migration in three-dimensional matrices. Biophys. J. 89, 1389–1397. (10.1529/biophysj.105.060723) 15908579 PMC1366623

[38] McDougall S Dallon J Sherratt J Maini P 2006 Fibroblast migration and collagen deposition during dermal wound healing: mathematical modelling and clinical implications. Phil. Trans. R. Soc. A 364, 1385–1405. (10.1098/rsta.2006.1773) 16766351

[39] Hawkins RJ Piel M Faure-Andre G Lennon-Dumenil A Joanny J Prost J Voituriez R 2009 Pushing off the walls: a mechanism of cell motility in confinement. Phys. Rev. Lett. 102, 058103 (10.1103/PhysRevLett.102.058103) 19257561

[40] Danuser G Allard J Mogilner A 2013 Mathematical modeling of eukaryotic cell migration: insights beyond experiments. Annu. Rev. Cell Dev. Biol. 29, 501–528. (10.1146/annurev-cellbio-101512-122308) 23909278 PMC4148455

[41] Neilson M Veltman DM van Haastert PJM Webb SD Mackenzie JA Insall RH 2011 Chemotaxis: a feedback-based computational model robustly predicts multiple aspects of real cell behaviour. PLoS Biol. 9, e1000618 (10.1371/journal.pbio.1000618) 21610858 PMC3096608

[42] Marée A Jilkine A Dawes A Grieneisen VA Edelstein-Keshet L 2006 Polarization and movement of keratocytes: a multiscale modelling approach. Bull. Math. Biol. 68, 1169–1211. (10.1007/s11538-006-9131-7) 16794915

[43] Painter KJ 2009 Modelling cell migration strategies in the extracellular matrix. J. Math. Biol. 58, 511–543. (10.1007/s00285-008-0217-8) 18787826

