# Supplementary material for: Cost–benefit analysis of the mechanisms that enable migrating cells to sustain motility upon changes in matrix environments
Source: J R Soc Interface. 2015 May 6;12(106):20141355. doi: 10.1098/rsif.2014.1355 (PMC4424668; doi:10.1098/rsif.2014.1355)
Supplement: Supplementary Information [file rsif20141355supp1.pdf]

Supplementary Methods

A detailed description of the cell motility model

There have been numerous studies investigating the factors determining the efficacy of cell motility, and probing the dynamics of underlying processes. The mechanisms behind different protrusion types are investigated with models of single bleb formation [28-32], and actin polymerisation dynamics [33-35]. Cell-matrix interactions are modelled with defining the ECM fibres explicitly or as a continuum density [36-40]. While excluding cell shape changes, these models provide insights on the influence of global cytoskeletal dynamics, adhesion requirements, and ECM fibre structures on cell motility. However, these models and others that include cell shape changes, do not explore the possibility of multiple modes of cell motility [41,42] and the description of multiple motility modes with pre-defined assumptions on cell-ECM interactions do not allow for investigation of emergent cell behaviour [43].

We share the goal of previous studies that aimed to identify the efficiencies of different motility modes [43], and the discretisation of the cell surface places our model closer to previous pseudopod based motility models [41]. Our model differs by the inclusion of multiple protrusion types, and emergent nature of motility modes.

In our computational model of single cell motility, the cell surface is discretized as a set of nodes with viscoelastic linkers in between. Each of the viscoelastic linkers is composed of a spring for myosin contractility, a spring for membrane tension, and a spring-dashpot combination (Kelvin-Voigt body) for the viscoelastic response of the actin network, all connected in parallel. The physical properties of the actin and myosin linkers are determined by the local protein concentrations at the linked nodes, while the membrane tension is a global property of the cell. Internal cell body responds to the deviations from cell's ideal size with changes in internal cell pressure, and applies a viscous response to the movement of the cell surface. The nucleus of the cell is composed of a nuclear lamina, similar in construction to the actin cortex of the cell surface, and a

viscoelastic interior, similar to the rest of the internal cell body. The nucleus is stiffer, and more viscous than the rest of the cell interior.

The protrusion formation is closely linked with both the current physical state of the cell body, and the protein concentration of the cell surface. Lamellipodia formation rate depends on local myosin concentration, mimicking the inhibitory cross talk between Rho and Rac. It should be noted the model is in 2D, and does not support the distinction between lamellipod, filopod, or a pseudopod in terms of their respective 3D structures. The behaviour of the actin polymerisation based protrusions is modelled to resemble lamellipodia behaviour, and as such, the terminology "lamellipodia" is used. The blebs are formed with the interplay between the intracellular pressure, and local ERM concentration linking the plasma membrane to the actin cortex: when local fluctuations of the ERM levels bring the cortex-membrane adhesion strength below the current intracellular pressure, the membrane detaches from the cortex, forming a new bleb [15].

The ECM filaments are defined explicitly, allowing for investigation of varying ECM topologies. We have detailed modelling for the adhesion to the ECM, and physical interactions with the fibres in terms volume exclusion. We do not focus on direct friction based mechanisms, which have been suggested as effective motility modes under confinement [38], and this could provide tolerance to zero adhesion levels under confined continuous environments.

The model is parameterised against experimental data on A375 human melanoma cells. Further details of the modelling technique, together with the experimental data and fitting procedures can be found in our previous publication [2]. The protein levels of the model are scaled to different cell types and subtypes based on experimental measurements of proteins relative to the A375P subtype used in parameterisation [2]. The overall cell contractility level at 1.4 combined with an ERM level of 1.0 represents A375M2 subtype, while the simulations with lower cell contractility (1.0 compared to 1.4) and low ERM levels (0.75 compared to 1.0) are closer to MDA-MB-231 breast cancer cells.

The polarity is defined as a shift of myosin content (Figure S1G). In-line with experimental observations (Figure S1E-F), in all the simulations, the cell "rear" is defined as the region with high myosin concentration.

We also have directly tested scenarios where the cells are polarised in lamellipodia formation without myosin polarity (Figure S1H). Under the tested conditions, polarity in lamellipodia formation without polarisation of myosin is not sufficient to give consistent directionality to the cell. Typically, the cells form lamellipodia in both directions, albeit with lower initiation rates on one side. In the absence of polarised contractile forces (myosin polarity), the cell can neither terminate the protrusions towards the "wrong" direction, nor detach its "rear" consistently. Thus, although the cells can still generate considerable movement, it is in arbitrary directions (Figure S1Hii/v). In an average of multiple simulations, on unconfined surfaces, the cells cannot generate movement, due to detachment at low adhesions (Adhesion level 5), and 'noise' at high adhesion levels (Figure S1Hii). Under confinement with high adhesion, the mean velocities are at the same levels as to the environments where lamellipodia are of little effect, such as discontinuous environments, or low adhesion (Figure S1H, compare panel v with iii&iv). Our results do not eliminate possibility of alternative contractility-independent mechanisms that terminate the 'un-desired' lamellipodia and disassemble cell-ECM adhesions at the cell rear. Such mechanisms would permit directional movement of the cell.

In this work, the density of agents defining the cell is reduced for faster simulations. Parameter fitting procedure is the same as detailed in our previous study (Figure S7). The resulting parameters are the same as given in Table S1 of Tozluoğlu *et. al.*, 2013; except for internal cytoskeleton viscosity, η_{CELL}, set to $3\times10^{-4}\mu N\ s\ \mu m^{-2}$, nucleus viscosity, η_{NUC}, set to $6\times10^{-4}\mu N\ s\ \mu m^{-2}$, adhesion force per unit cortex-membrane linker protein set to 120 pN, and lamellipodia formation rate at 1.0 myosin level, k_f^0, set to 0.01 sec^{-1}.

Initiation of blebs on retracting blebs

Layered bleb formations are frequently observed in cancer cells. We improve our model by redefining plasma membrane blebbing to allow the formation of new blebs on top of retracting blebs. This leads to the formation of multi-layered blebs, with multiple decaying bleb necks of different stiffness (Figure S1B-C), similar to those observed in cancer cells (Figure S1D).

In the model, actin cortex-plasma membrane linker protein levels stochastically fluctuate, via an experimentally measured probability distribution, around a mean value defined by cell polarity [2]. Plasma membrane blebs are formed when forces exerted by the internal hydrostatic pressure of the cell exceeds the cortex-membrane attachment strength at a weaker point of the cell surface [15]. Mimicking biological behaviour of blebs, the model blebs go through a rapid expansion, a stabilisation, and a slow retraction where actin re-accumulates at the bleb rim. In our previous model, the cell surface belonging to a bleb would become available for a new bleb initiation only once the bleb is fully retracted, the blebbing cell surface is incorporated to the regular cell surface, and the residual cortex at the bleb neck is fully decayed [2]. In the current model, initiation of new blebs upon retracting blebs are allowed, once the actin content of the retracting bleb rim reached 50 per cent of regular cell surface. Eventually, depending on the dynamics of pressure and cortex-membrane linkers, multi-layered blebs emerge, with sequentially accumulated bleb necks forming ladder like structures (Figure S1B-C). Decay of the bleb necks, and re-accumulation of the actomyosin cortex at bleb rims remaining between these neck layers continue at each bleb's own rate.

Protrusion scores

Protrusion data is collected at 1-second intervals during each simulation. Spreading lamellipodia score is defined separately for lamellipodia spreading towards the cell rear and cell front, cell rear defined as the side with increased myosin concentration. The spreading lamellipodia score is binary, with the value of one if there is at least one spreading lamellipodia towards the selected direction, and zero otherwise. (The actual number of spreading lamellipodia on

one side can be maximum one for an unconfined surface, and two within confined continuous environments. The number can be higher in complex *in-vivo* mimetic environments.) These scores are binned and averaged over simulations with respect to the position of cell centre at same time point. Therefore, under any given condition, the frontal or rear spreading lamellipodia score can take a value between 0 and 1.

The blebbing score is defined as the percentage of the cell surface that is a part of a bleb at any data collection time point. While calculating the percentage, the entire surface belonging to a lamellipod is excluded from the calculation, to avoid biased lower percentages for blebbing surfaces when the cell has a long, thin spreading lamellipodia. Similar to lamellipodia scores, the blebbing scores are binned and averaged with respect to the position of the cell centre. The blebbing score can take any value between 0 and an unlikely level of 100 per cent.

Cell-ECM adhesion strengthening

The adhesion strengthening is modelled such that a given force applied on an adhesion point defines the equilibrium adhesion protein concentration at that junction. The current adhesion concentration is gradually updated towards the equilibrium adhesion concentration according to the equation

$$c_{CEA}(t+dt) = c_{CEA}(t) + \left(\frac{c_{CEA}^{eq}(t) - c_{CEA}(t)}{\tau_{CEA}}\right) \times dt. \qquad (1)$$

Here, $c_{CEA}(t)$ is the concentration of cell-ECM adhesion protein at a selected agent at time t; $c_{CEA}^{eq}(t)$ is the equilibrium adhesion concentration, induced by the forces applied on the same agent at time t; τ_{CEA} is the adhesion concentration renewal time of 3 minutes, and dt is the time step. The equilibrium adhesion concentration is related to the applied forces via equation:

$$c_{CEA}^{eq}(t) = \begin{cases} c_{CAE}^{min} & , if\ (p_{\parallel} \times F_{\parallel} + p_{\perp} \times F_{\perp}) \leq F_{min} \\ c_{CEA}^{max} & , if\ (p_{\parallel} \times F_{\parallel} + p_{\perp} \times F_{\perp}) \geq F_{max} \\ c_{CAE}^{min} + \left((p_{\parallel} \times F_{\parallel} + p_{\perp} \times F_{\perp}) - F_{min}\right) \times \frac{\left(c_{CEA}^{max} - c_{CEA}^{min}\right)}{(F_{max} - F_{min})} & , else \end{cases} . \qquad (2)$$

Here, c_{CAE}^{min} is the initial adhesion at a contact site, it defines the minimum strength any given adhesion will be at, regardless of the forces applied; c_{CEA}^{max} is the maximum adhesion concentration that can be achieved within a single contact zone. F_{min} is the lower limit of mechanosensing, above which the cell starts reinforcing the adhesion, and F_{max} is the force magnitude that induces the maximum adhesion cell could reach (Figure 5A). The subscripts ∥ and ⊥ denote parallel and perpendicular orientations with respect to the surface of adhesion, F is the current force applied on the adhesion site, and p are the weighing factors for shear and perpendicular force components. During parameter search, among the perpendicular forces, those pushing the cell into the filament do not reinforce adhesions, and only pulling forces are considered in the calculation. Both up and down regulation of adhesion strength are regulated by the same rules. Finally, upon detachment, the adhesion protein concentration at any site is reset to c_{CAE}^{min}.

Within the tested parameter range (Figure S5), the following set is identified to be the most beneficial for the cell: $p_{\parallel} = 1.0, p_{\perp} = 0.0$, $c_{CAE}^{min} = 5$, $c_{CAE}^{max} = 250$, F_{min} = 0.0 pN, and $F_{max} = 300\ pN$. In simulations where the adhesion strength is dictated by the environment, the low ligand concentration zones are defined by the above parameters, and high ligand concentration zones are defined with $c_{CAE}^{min} = 20$ and $F_{min} = 18.37$ pN, with the remainder of parameters being the same.

Supplementary Figure Legends

Supplementary Figure 1. The single cell motility model setup. A) Schematic representation of modelling methodology and blebbing on top of retracting blebs. B) Simulation snapshots from a model cell moving through a discontinuous series of fibres, cell myosin level, 1.4, 50% increased contractility at the cell rear accompanied by a 50% reduction of contractility at cell front, and 40% decreased ERM levels at cell front. Scale bar 10 microns. C) Simulation snapshots from a series of blebs forming sequentially on top of each other, the residual cortex from older blebs can be seen. Cell conditions same as (B), scale bar 2 microns. D) Maximum intensity projection of phalloidin (f-actin) stained MDA-MB-231 human breast cancer cell forming blebs on top of existing blebs, scale bar 5 microns. Inset is showing a single confocal cross-section displaying the layers of blebs. E) Two MDA-MB-231 cells *in vitro*, exemplifying the polarity of myosin. i/v) phalloidin (F-actin) staining (blue) ii/vi) myosinIIA staining (red), iii/vii) Ezrin staining (green), and iv/viii) merge. F) Quantification of experimental data. Intensity of f-actin (blue), myosin (red), and ezrin (green) staining with respect to the normalised distance from cell rear, an average of 5 cells as those represented in (E). G) Definition of myosin polarity in the model, i) the overall myosin levels are conserved; a 50% increase at the cell rear induces a 50% reduction of myosin levels at cell front. ii) Schematic showing the normalised distance definition in (i). H) Performance of a cell with no myosin polarity, but with increased lamellipodia formation propensity at front and reduced lamellipodia formation at rear, equivalent to that induced by 50% myosin polarity. The cell has 40% reduced ERM at cell front, cell myosin level is 1.4. i) Velocity heatmap, adhesion levels are indicated on the y-axis. x-axis represent environment geometries, from left to right, on unconfined surface, continuous confined environment, and discontinuous confined environment. ii-v) Scatter plots for velocities of individual simulations ($\mu m\ min^{-1}$), horizontal blue line marks the mean value, and vertical black line marks one standard deviation. ii) Unconfined surface at adhesion level of 5, iii) discontinuous confined environment at adhesion 5, iv) continuous confined environment at adhesion 5, and v) continuous confined environment at adhesion 30.

Supplementary Figure 2. Performance of restricted motility modes. A) Velocity heatmaps showing the performance of a cell with only lamellipodia (top row) and with only plasma membrane blebbing (bottom row), i) on a surface, ii) in a continuous confined environment, and iii) in a discontinuous confined environment. Cell has 50% increased contractility at the cell rear accompanied by a 50% reduction of contractility at cell front, and 40% decreased ERM levels at cell front. Adhesion levels are indicated on the y-axis, and overall cell contractility is on the x-axis. The velocity is in μm min^{-1}. Colourbar valid for i-iii. B) Front spreading lamellipodia scores for the cell with only lamellipodia formation (data corresponding to A, top row), data representation same as in (A). C) Blebbing surface percentage scores for cell with only bleb formation (data corresponding to A, bottom row), data representation same as in (A).

Supplementary Figure 3. Influence of adhesion and cell type on motility mode shift. A-B) Cell has 50% increased contractility at the cell rear accompanied by a 50% reduction of contractility at cell front, and 40% decreased ERM levels at cell front. Cell – ECM adhesion level at 20 units. Data points taken every second of simulation time; binned at 1 μm intervals with respect to the position of cell centre. All plots averaged over at least 10 simulations. i) Environment schematic, ii) intracellular pressure (Pa). Pressure data presented in (Aii) and (Bii) complement the data on Figure 2A and 2B, respectively. C) Cell moving from a confined continuous environment to an unconfined surface; D) from an unconfined surface to a continuous confined environment, and back to the unconfined surface. For (C) and (D), cell polarity is same as in (A), overall myosin level is 1.4, and cell-ECM adhesion is 10 units. Data measurement and binning is the same as (A) i) Environment schematic, ii) Instantaneous cell velocity (μm min^{-1}), iii) Front spreading lamellipodia score (green), rear spreading lamellipodia score (dashed red), and percentage of blebbing cell surface (blue) plotted as a function of cell centre position within the environment. iv) Internal pressure of the cell (Pa) v) Statistical analysis of average protrusion scores for the position intervals marked on the environment schematic (i). Two-tail t-test carried out between scores of each interval within

the environment, “*” indicates p-value below 0.05, “**” below 0.01, and “***” below 0.001. E-F) Cell properties, data binning and representation are same as in (A). Pressure data presented in (Eii) and (Fii) complement the data on Figure 2C, and 2D, respectively. G-J) Environment transition profiles for a cell adapted to a more lamellipodia based motility mode, mimicking MDA-MB-231 breast cancer cells. Relative to the cells closer to A375M2 human melanoma cells used throughout rest of the simulations of the paper, the cells have lower contractility (1.0 compared 1.4), lower membrane-cortex adhesion (0.75 compared to 1.0), and higher cell-ECM adhesion (25 compared to 20). Data representation and binning is the same as in Figure 2A.

Supplementary Figure 4. Performance in changing ECM geometries at higher polarities and variable myosin – ERM localisation. Data structure and representation is same as in Figure 2A. Cell overall myosin is 1.4, adhesion to ECM is 20 units. A-D) Cells are polarised with 70% increased myosin at the cell rear, accompanied by 70% reduction at cell front, and 60% reduced ERM at cell front. E-H) Myosin polarity is same as in (A-D), with 60% reduced ERM at cell rear.

Supplementary Figure 5. Tension-adhesion feedback mechanism parameter search. Each panel show a different combination of adhesion range available to the cell ($c_{CAE}^{min} - c_{CAE}^{max}$), and the force range of mechanosensing ($F_{min} - F_{max}$). (A-B) Varying ($c_{CAE}^{min} - c_{CAE}^{max}$) range where all positive forces induce strengthening ($F_{min} = 0$), (C-D) Varying ($c_{CAE}^{min} - c_{CAE}^{max}$) range where cells sense forces above 50 pN ($F_{min} = 50$). Each inset (i) shows the corresponding tension-adhesion feedback curve, insets (ii-iv) show cell velocity as a function of adhesion for the force directions cells utilise in the feedback ($p_{\parallel}, p_{\perp}$). Left plot, for cell velocity on an unconfined surface, right plot for confined discontinuous environment. Cell behaviour with (solid lines) and without (dashed lines) mechanosensing, are plotted together for comparison. Red is for overall contractility set to 0.7 and green is for 1.4. Cell polarity is the same as in Figure 4A. A) $c_{CAE}^{min} = 5, c_{CAE}^{max} = 150, F_{min} =$ 0.0 pN, and $F_{max} = 300$ pN. B) $c_{CAE}^{min} = 5, c_{CAE}^{max} = 250, F_{min} =$ 0.0 pN, and $F_{max} = 300$ pN. C) $c_{CAE}^{min} = 5$,

c_{CAE}^{max} = 150, F_{min} = 50.0 pN, and F_{max} = 300 pN. D) c_{CAE}^{min} = 5, c_{CAE}^{max} = 250, F_{min} = 50.0 pN, and F_{max} = 300 pN. In each panel, insets (ii) utilise perpendicular, pulling forces only, with no adhesion strengthening via shear forces ($p_{\parallel}$ = 0.0, $p_{\perp}$ = 1.0), (iii) utilise both perpendicular, pulling and shear forces with 0.5 weighing factors ($p_{\parallel}$ = 0.5, $p_{\perp}$ = 0.5), and (iv) utilise shear forces only, with no influence form perpendicular forces ($p_{\parallel}$ = 1.0, $p_{\perp}$ = 0.0). See Methods - Cell-ECM adhesion strengthening for interpretation of parameters. Inset (Biv) plots the same data as Figure 5C-D.

Supplementary Figure 6. Influence of mechanosensing on adhesion levels and cell motility plasticity. A) Spatial distributions of adhesion points above selected thresholds. i) The schematic representation of how the data is generated. The distance of each adhesion point is calculated relative to the cell centre. The histograms are constructed to represent the distribution of the adhesion number, with respect to the distance from cell centre. For each histogram, adhesions only above the selected threshold are counted. ii-iii) Histograms for a cell moving on a surface. The thresholds are 30 units for cyan, and 100 for black. Y- axis is normalised with the total number of adhesions. X-axis is the distance from cell centre. ii) The shear forces, pulling the agent on the axis parallel to the surface enable adhesion strengthening. iii) The pulling forces perpendicular to the surface enable adhesion strengthening. B)Distribution of adhesions on a spreading cell, i) quantification of the labelling, the adhesions are high at the front and rear regions of the cell. ii) The image where data in (i) is obtained, C-D) These insets form the counterparts of Figure 6, with the high/low adhesion zones defined in inverse order, panels (iii). All cell parameters and adhesion behaviour is the same as Figure 6. Cv) Instantaneous velocity for a cell with mechanosensing, at lower contractility (1.0 compared 1.4 in (iv)), lower membrane-cortex adhesion (0.75 compared to 1.0 in (iv)). All average of at least 10 simulations.

Supplementary Figure 7. Parameterisation of the single cell motility model. A) i-iv) Heatmaps of parameter fitting score. x-axis shows the increasing inner cell body (cytoskeleton) viscosity. y-axis demonstrates the initial actin fraction

that will be left at the detaching cortex, at the onset of bleb initiation. Each heatmap presents fitting scores at different cell cortex-plasma membrane adhesion strength per unit protein, the values are indicated in the titles. Bleb dynamics are fitted to experimental data for each parameter set. The plotted score is the mean of R^2 fit for maximum bleb size, bleb expansion rate, and bleb retraction rates. The selected parameter set in boxed in Aiv. v) Colourbar is valid for (Ai-iv). B) Spreading lamellipodia score for resting cells with overall contractility of 1.0, as a function of lamellipodia initiation rate constant k_f^0 . The experimental measurement is plotted as a red line [2], the selected parameter marked by “*”.

Supplementary Movie Legends

Supplementary Movie 1. Environment change from an unconfined surface to a confined discontinuous environment. Overall cell contractility set to 1.4, cell-ECM adhesion fixed at 20 units, 50 per cent increased myosin at cell rear accompanied by 50 per cent myosin reduction at cell front, and 40 per cent ERM reduction at cell front.

Supplementary Movie 2. Environment change in between confined and unconfined surfaces. Environment is changing from an unconfined surface to a confined continuous geometry, and back into an unconfined surface. Overall cell contractility set to 1.4, cell-ECM adhesion fixed at 20 units, 50 per cent increased myosin at cell rear accompanied by 50 per cent myosin reduction at cell front, and 40 per cent ERM reduction at cell front.

Supplementary Movie 3. Environment change from an unconfined surface to an *in vivo* mimetic matrix. Overall cell contractility set to 1.4, cell-ECM adhesion fixed at 20 units, 50 per cent increased myosin at cell rear accompanied by 50 per cent myosin reduction at cell front, and 40 per cent ERM reduction at cell front. Cell polarity is realigned during simulation, defining cell front towards the tip point of the cell in the (+)ve x direction [2].

Supplementary Movie 4. Environment change in cells with co-localised myosin and ERM polarity. Overall cell contractility set to 1.4, cell-ECM adhesion fixed at 20 units, 50 per cent increased myosin at cell rear accompanied by 50 per cent myosin reduction at cell front, and 40 per cent ERM reduction at cell rear. i) Environment is changing from an unconfined surface to a confined discontinuous environment. ii) Environment is changing from an unconfined surface to a confined continuous geometry, and back into an unconfined surface.

Supplementary Movie 5. Influence of mechanosensing upon environment geometry and adhesion changes. i) Movement of a cell with mechanosensing ability is displayed, in an environment with changing adhesion and geometry, from an unconfined surface, to a confined discontinuous geometry. ii) Same

environment with the adhesions fixed, as dictated by the ECM. Overall cell contractility set to 1.4, 50 per cent increased myosin at cell rear accompanied by 50 per cent myosin reduction at cell front, and 40 per cent ERM reduction at cell front. iii) Close view of (i), and iv) close view of (ii).

Supplementary Movie 6. Influence of mechanosensing. i) Movement of a cell with mechanosensing ability is displayed, in an environment with changing adhesion and geometry, from a confined discontinuous geometry, to an unconfined surface. ii) Same environment with the adhesions fixed, as dictated by the ECM. Overall cell contractility set to 1.4, 50 per cent increased myosin at cell rear accompanied by 50 per cent myosin reduction at cell front, and 40 per cent ERM reduction at cell front.
