# Supplementary material for: Cost–benefit analysis of the mechanisms that enable migrating cells to sustain motility upon changes in matrix environments
Source: J R Soc Interface. 2015 May 6;12(106):20141355. doi: 10.1098/rsif.2014.1355 (PMC4424668; doi:10.1098/rsif.2014.1355)
Supplement: Supplementary Figures [file rsif20141355supp2.pdf]

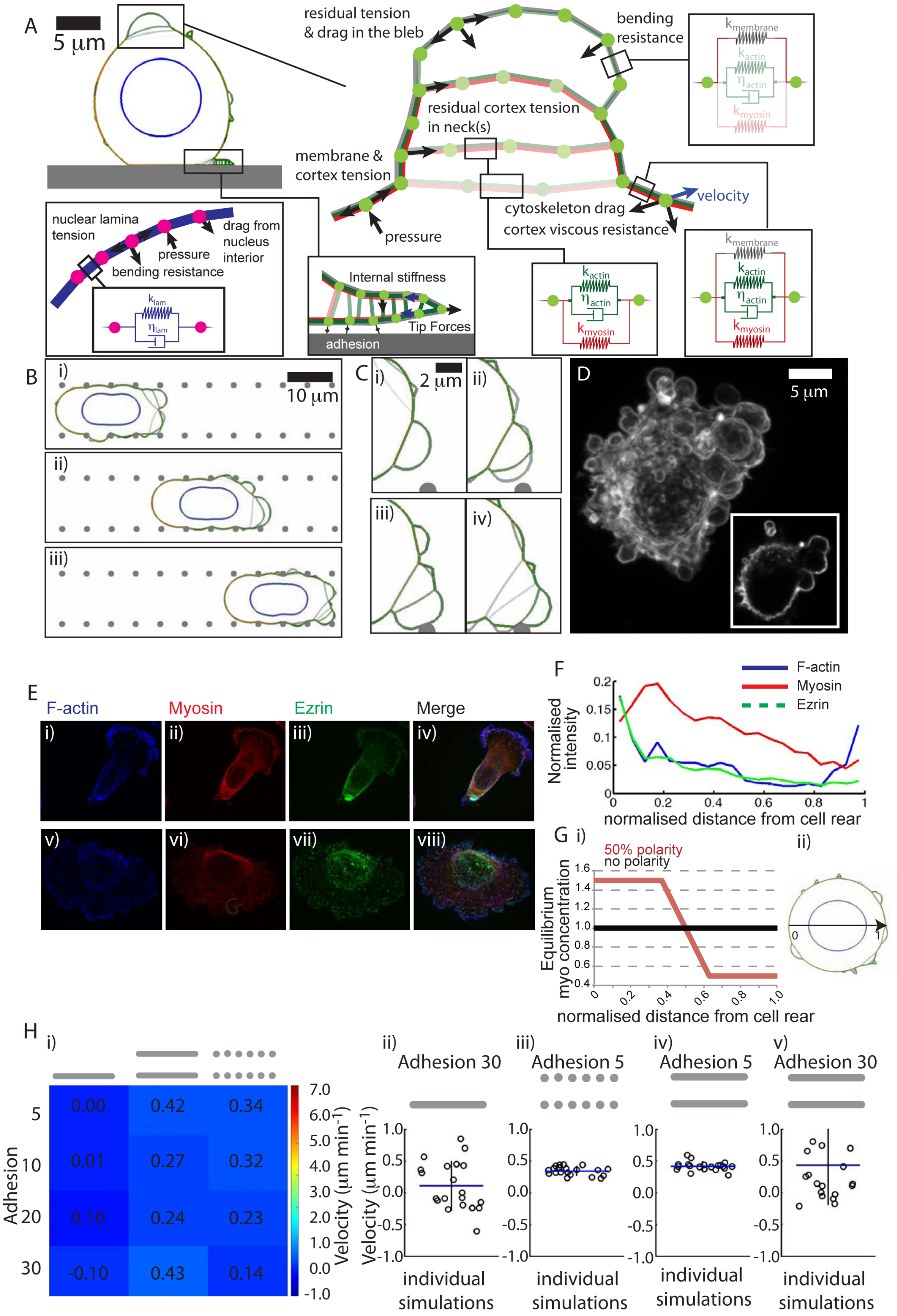
A
5 µm
residual tension
& drag in the bleb
bending
resistance
k membrane
k actin
η actin
k myosin
residual cortex tension
in neck(s)
membrane &
cortex tension
velocity
cytoskeleton drag
cortex viscous resistance
pressure
nuclear lamina
tension
drag from
nucleus
interior
pressure
bending resistance
k lam
η lam
Internal stiffness
Tip Forces
adhesion
B
i)
10 µm
ii)
iii)
C
i)
2 µm
ii)
iii)
iv)
D
5 µm
E
F-actin
Myosin
Ezrin
Merge
i)
ii)
iii)
iv)
v)
vi)
vii)
viii)
F
F-actin
Myosin
Ezrin
Normalised
intensity
normalised distance from cell rear
G i)
50% polarity
no polarity
Equilibrium
myo concentration
normalised distance from cell rear
ii)
H
i)
Adhesion
5
10
20
30
0.00
0.42
0.34
0.01
0.27
0.32
0.10
0.24
0.23
-0.10
0.43
0.14
Velocity (µm min⁻¹)
ii)
Adhesion 30
iii)
Adhesion 5
iv)
Adhesion 5
v)
Adhesion 30
Velocity (µm min⁻¹)
individual
simulations

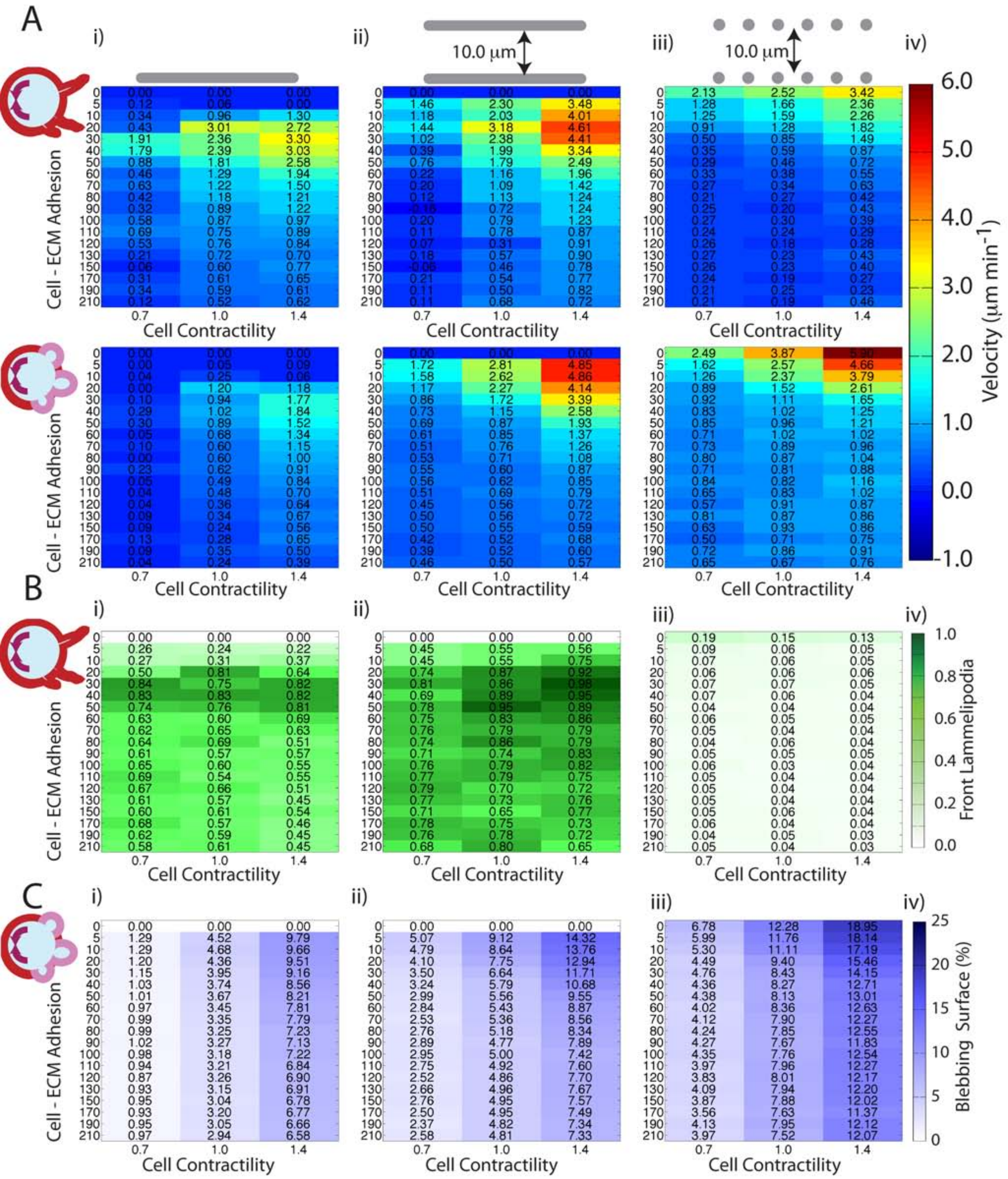

A
B
C
i)
ii)
iii)
iv)
10.0 μm
10.0 μm
Cell - ECM Adhesion
Cell Contractility
0.7
1.0
1.4
Velocity (μm min^{-1})
6.0
5.0
4.0
3.0
2.0
1.0
0.0
-1.0
Front Lammelipodia
1.0
0.8
0.6
0.4
0.2
0.0
Blebbing Surface (%)
25
20
15
10
5
0

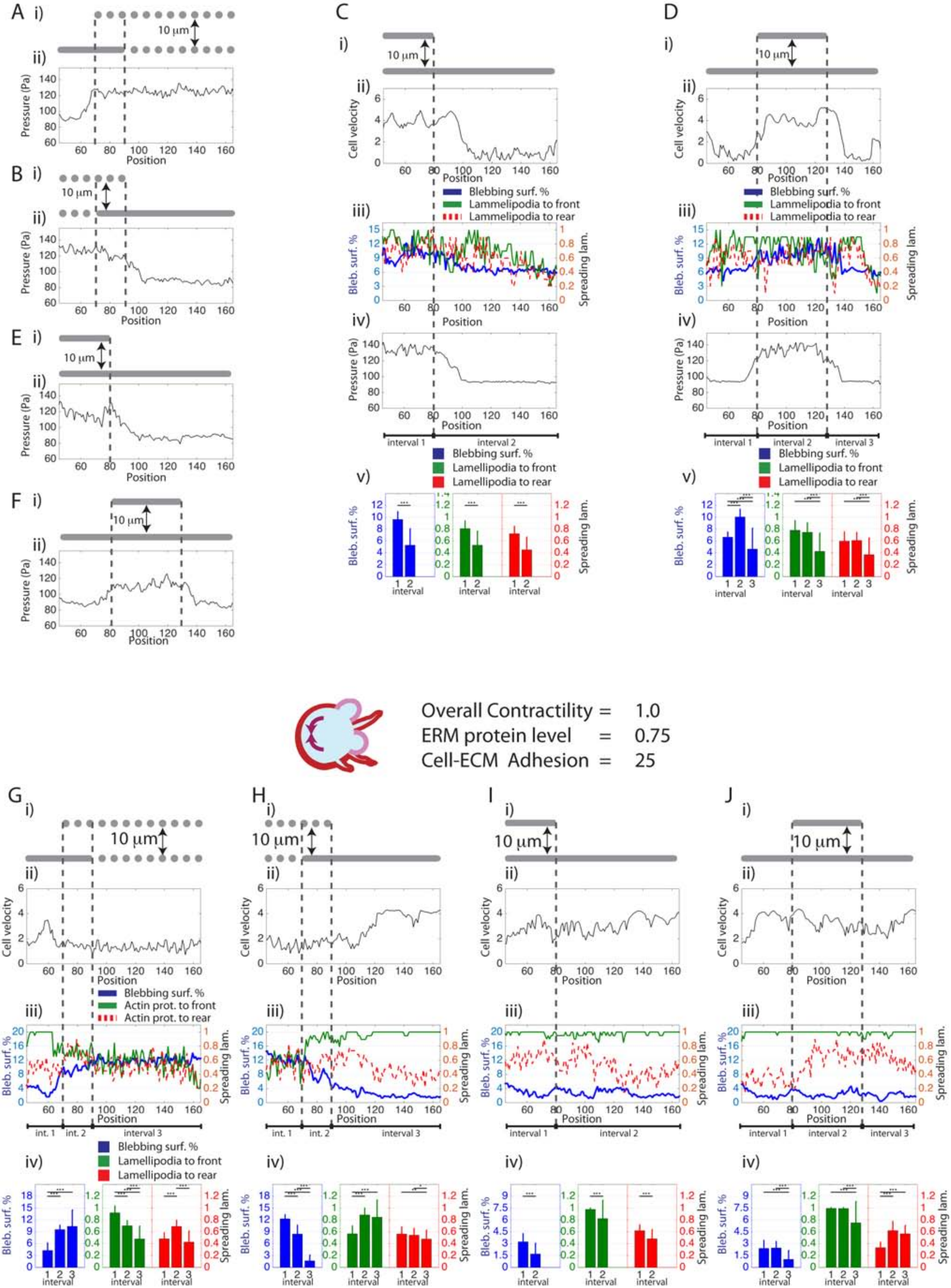

A
B
C
D
E
F
G
H
I
J
10 μm
Pressure (Pa)
Position
Cell velocity
Blebbing surf. %
Lammelipodia to front
Lammelipodia to rear
Bleb. surf. %
Spreading lam.
interval 1
interval 2
interval 3
Lamellipodia to front
Lamellipodia to rear
interval
Overall Contractility = 1.0
ERM protein level = 0.75
Cell-ECM Adhesion = 25
Actin prot. to front
Actin prot. to rear
int. 1
int. 2

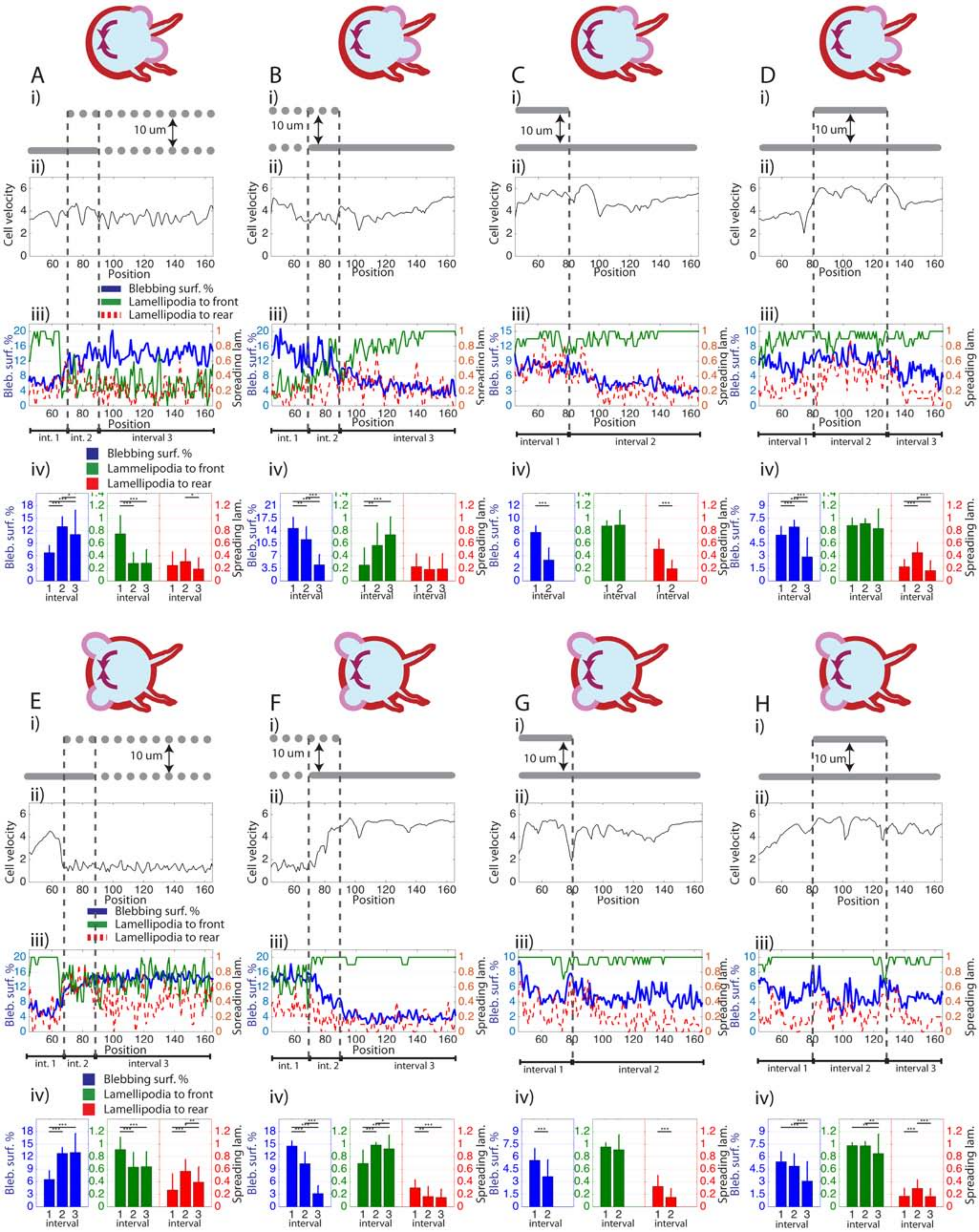

A
B
C
D
E
F
G
H
i)
ii)
iii)
iv)
10 um
Cell velocity
Position
Blebbing surf. %
Lamellipodia to front
Lamellipodia to rear
Lammelipodia to front
Bleb. surf. %
Spreading lam.
int. 1
int. 2
interval 1
interval 2
interval 3
interval

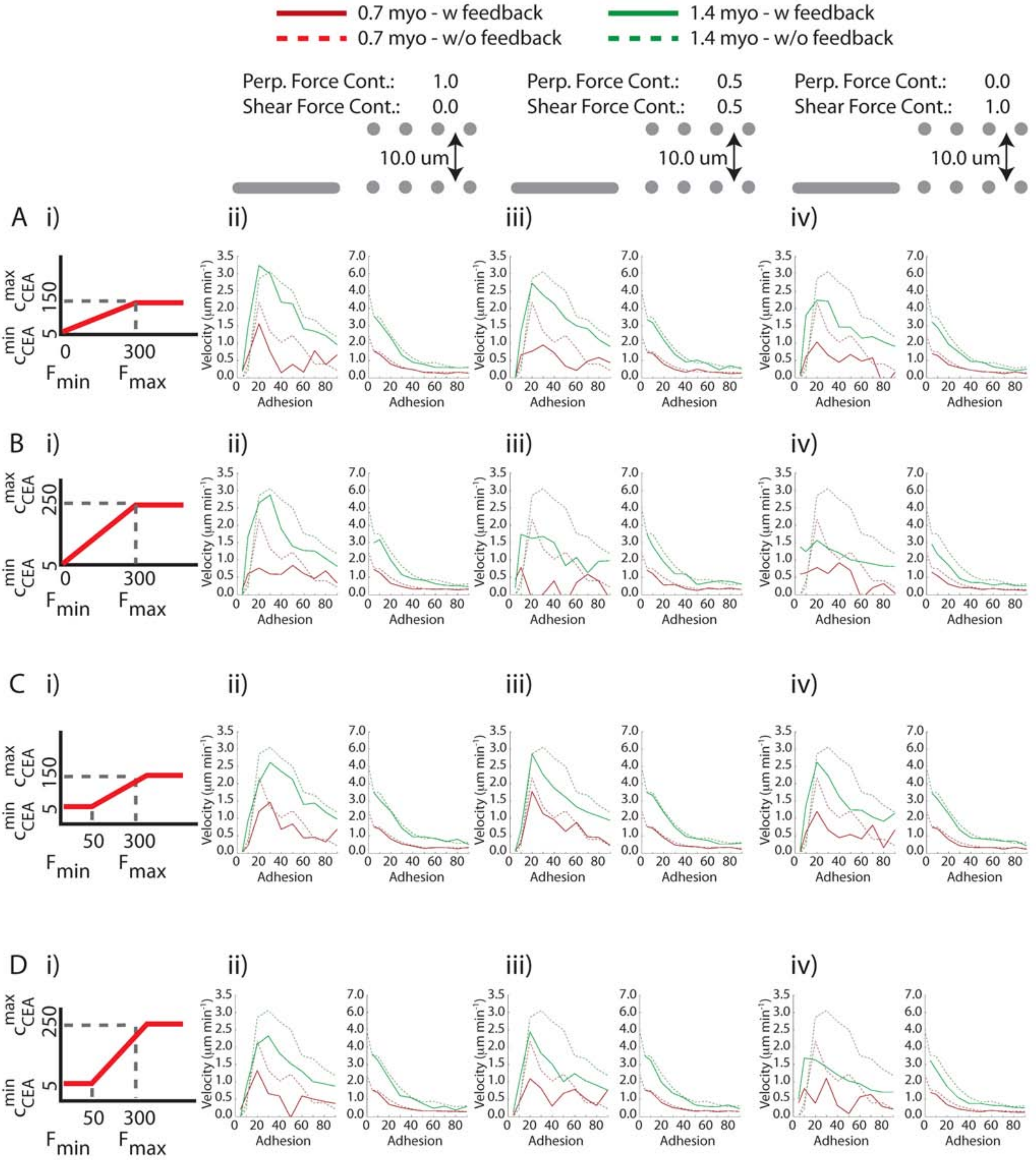

0.7 myo - w feedback
0.7 myo - w/o feedback
1.4 myo - w feedback
1.4 myo - w/o feedback
Perp. Force Cont.: 1.0
Shear Force Cont.: 0.0
Perp. Force Cont.: 0.5
Shear Force Cont.: 0.5
Perp. Force Cont.: 0.0
Shear Force Cont.: 1.0
10.0 um
A i) ii) iii) iv)
B i) ii) iii) iv)
C i) ii) iii) iv)
D i) ii) iii) iv)
c_{CEA}^{max}
c_{CEA}^{min}
F_{min}
F_{max}
Velocity (μm min⁻¹)
Adhesion

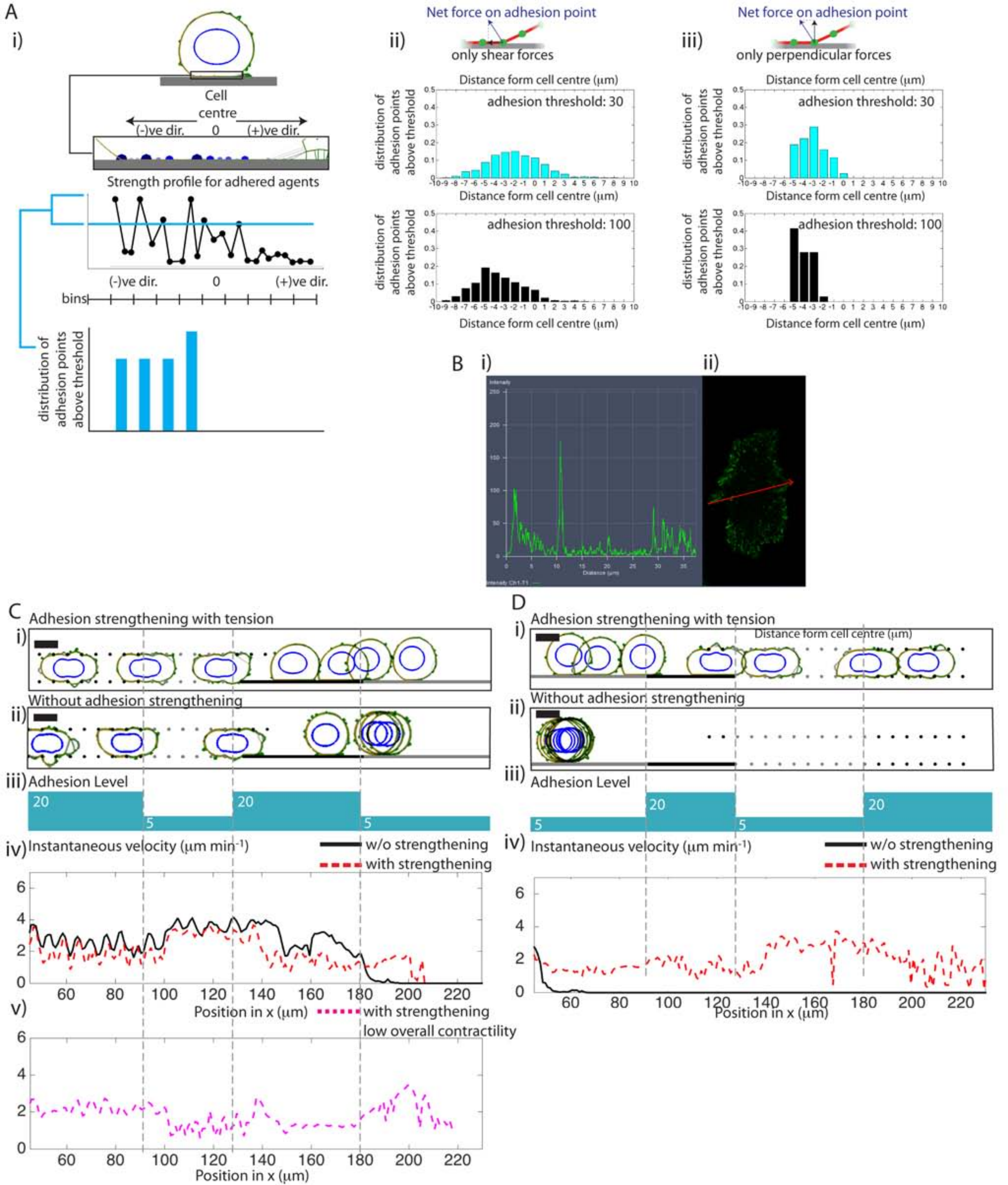

A
i)
Cell centre
(-)ve dir.
0
(+)ve dir.
Strength profile for adhered agents
(-)ve dir.
0
(+)ve dir.
bins
distribution of adhesion points above threshold
ii)
Net force on adhesion point
only shear forces
Distance form cell centre (μm)
adhesion threshold: 30
adhesion threshold: 100
distribution of adhesion points above threshold
iii)
Net force on adhesion point
only perpendicular forces
Distance form cell centre (μm)
adhesion threshold: 30
adhesion threshold: 100
B
i)
ii)
C
Adhesion strengthening with tension
i)
Without adhesion strengthening
ii)
iii) Adhesion Level
20
5
20
5
iv) Instantaneous velocity (μm min-1)
w/o strengthening
with strengthening
Position in x (μm)
v)
with strengthening low overall contractility
Position in x (μm)
D
Adhesion strengthening with tension
i)
Distance form cell centre (μm)
Without adhesion strengthening
ii)
iii) Adhesion Level
5
20
5
20
iv) Instantaneous velocity (μm min-1)
w/o strengthening
with strengthening
Position in x (μm)

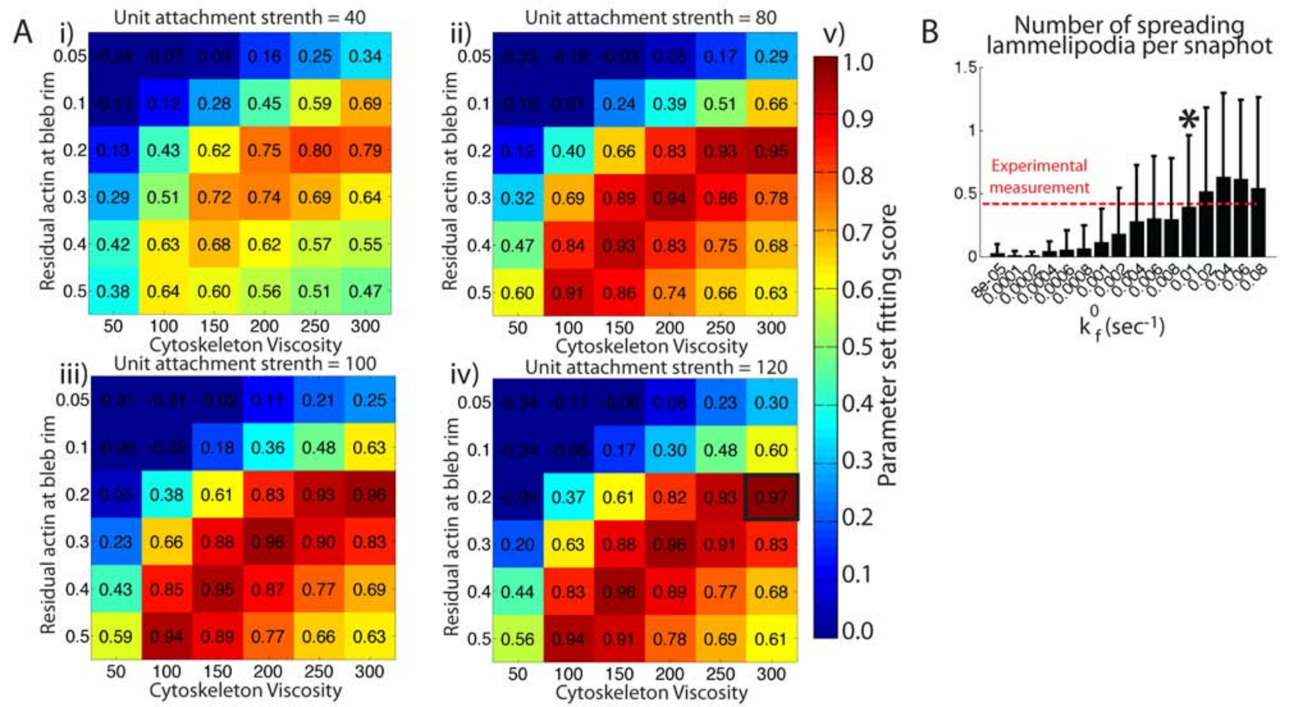
A
i)
Unit attachment strenth = 40
0.16 0.25 0.34
0.12 0.28 0.45 0.59 0.69
0.13 0.43 0.62 0.75 0.80 0.79
0.29 0.51 0.72 0.74 0.69 0.64
0.42 0.63 0.68 0.62 0.57 0.55
0.38 0.64 0.60 0.56 0.51 0.47
Residual actin at bleb rim
0.05 0.1 0.2 0.3 0.4 0.5
Cytoskeleton Viscosity
50 100 150 200 250 300
ii)
Unit attachment strenth = 80
0.17 0.29
0.24 0.39 0.51 0.66
0.40 0.66 0.83 0.93 0.95
0.32 0.69 0.89 0.94 0.86 0.78
0.47 0.84 0.93 0.83 0.75 0.68
0.60 0.91 0.86 0.74 0.66 0.63
iii)
Unit attachment strenth = 100
0.11 0.21 0.25
0.18 0.36 0.48 0.63
0.38 0.61 0.83 0.93 0.96
0.23 0.66 0.88 0.96 0.90 0.83
0.43 0.85 0.95 0.87 0.77 0.69
0.59 0.94 0.89 0.77 0.66 0.63
iv)
Unit attachment strenth = 120
0.23 0.30
0.17 0.30 0.48 0.60
0.37 0.61 0.82 0.93 0.97
0.20 0.63 0.88 0.96 0.91 0.83
0.44 0.83 0.96 0.89 0.77 0.68
0.56 0.94 0.91 0.78 0.69 0.61
v)
Parameter set fitting score
0.0 0.1 0.2 0.3 0.4 0.5 0.6 0.7 0.8 0.9 1.0
B
Number of spreading lammelipodia per snaphot
Experimental measurement
0 0.5 1 1.5
*
8e-05 0.0001 0.0002 0.0004 0.0006 0.0008 0.001 0.002 0.004 0.006 0.008 0.01 0.02 0.04 0.06 0.08
k_f^0 (sec^{-1})
